# From ground pools to treeholes: convergent evolution of habitat and phenotype in *Aedes* mosquitoes

**DOI:** 10.1186/s12862-017-1092-y

**Published:** 2017-12-19

**Authors:** John Soghigian, Theodore G. Andreadis, Todd P. Livdahl

**Affiliations:** 1 Center for Vector Biology & Zoonotic Diseases, The Connecticut Agricultural Experiment Station, 123 Huntington St., New Haven, CT 06511 USA; 2 Biology Department, Clark University, 950 Main Street, Worcester, MA 01610 USA

**Keywords:** Convergence, Evolution, Habitat specialization, Aedes, Mosquitoes

## Abstract

**Background:**

Invasive mosquito species are responsible for millions of vector-borne disease cases annually. The global invasive success of *Aedes* mosquitoes such as *Aedes aegypti* and *Aedes albopictus* has relied on the human transport of immature stages in container habitats. However, despite the importance of these mosquitoes and this ecological specialization to their widespread dispersal, evolution of habitat specialization in this group has remained largely unstudied. We use comparative methods to evaluate the evolution of habitat specialization and its potential influence on larval morphology, and evaluate whether container dwelling and invasiveness are monophyletic in *Aedes*.

**Results:**

We show that habitat specialization has evolved repeatedly from ancestral ground pool usage to specialization in container habitats. Furthermore, we find that larval morphological scores are significantly associated with larval habitat when accounting for evolutionary relationships. We find that Ornstein-Uhleinbeck models with unique optima for each larval habitat type are preferred over several other models based predominantly on neutral processes, and that OU models can reliably simulate real morphological data.

**Conclusions:**

Our results demonstrate that multiple lineages of *Aedes* have convergently evolved a key trait associated with invasive success: the use of container habitats for immature stages. Moreover, our results demonstrate convergence in morphological characteristics as well, and suggest a role of adaptation to habitat specialization in driving phenotypic diversity in this mosquito lineage. Finally, our results highlight that the genus *Aedes* is not monophyletic.

**Electronic supplementary material:**

The online version of this article (10.1186/s12862-017-1092-y) contains supplementary material, which is available to authorized users.

## Background

Invasive mosquito vectors account for more than 100 million clinical disease cases annually [1]. The global invasive success of vector mosquito species has relied on the utilization of domestic containers for immature stages, which can be transported incidentally by human trade [2–5]. Invasive species from the genus *Aedes* have been found on six continents, are frequently dispersed by human activities, and represent a serious threat to public health due to their ability to transmit numerous human pathogens. All such invasive *Aedes* utilize containers, natural or domestic, for immature stages, and all are thought to have become established outside native ranges due to human-aided dispersal [3, 6–9]. Despite the importance of container dwelling larvae to the invasive success of these mosquitoes, it is largely unknown whether container dwelling – and thus invasiveness – has evolved multiple times in this genus, or if instead, invasive *Aedes* mosquitoes share a common, container dwelling ancestor.

Mosquitoes (Diptera: Culicidae) are a major radiation of true flies, encompassing more than 3500 species. The largest tribe of mosquitoes is the Aedini, a clade of mosquitoes over 100 million years old [10] with 1255 species in 10 genera [6], the most notable of which is the genus *Aedes*, presently recognized as containing 929 species [6] (Additional file 1: Table S1). Aedine mosquitoes are globally distributed and many species are important vectors of human or veterinary disease [6], such as Dengue, chikungunya and Zika virus [1]. Although the Aedini are best known for container dwelling invasive disease vector species such as *Aedes aegypti* and *Aedes albopictus*, the tribe is a phenotypically diverse group of mosquitoes utilizing many larval habitats, such as temporary ground pools, temporary salt pools, rock pools, as well as container habitats (Table 1).

**Table 1:** Common larval habitats of the Aedini

Habitat Type	N^a^	Descriptions and/or examples
Container	117 (16)	Small fresh water habitats made from decaying holes in trees or stumps, water holding leaf axils, fallen fronds and coconut shells, small rock holes
Rock Pools	20 (16)	Temporary fresh water habitat held by rock surfaces, sometimes along streams or rivers, or in caves.
Crab Hole	4	Small holes in mud flats or salt marshes created by crabs or other invertebrates, filled with salt water
Salt Pool	23 (7)	Variable salinity habitats that are flooded due to tidal action or wave splashing, including salt marshes or earthen depressions flooded by salt water
Ground Pool	118 (9)	Fresh water habitats that experience periodic inundation from precipitation, melting snow, or flooding; flood plains, temporary or semi-permanent swampland, snow melt pools, or muddy fields where depressions collect water.

^a^N is the total number of taxa for a given habitat specialization in this study. (N) indicates number of species utilizing this habitat as well as another

Of the five *Aedes* mosquito species considered invasive worldwide [3, 6–9], all develop as larvae within aquatic container habitats, suggesting the importance of this trait in facilitating the long-range dispersal and invasive success of these species. Container dwelling mosquitoes have desiccation resistant eggs that are typically deposited just above the water’s surface on the sides of small containers [4], making them particularly apt for dispersal when laid in domestic containers such as used tires, flower pots and buckets. Hatching habitually occurs when eggs are inundated following a rainfall event. Species that occupy container habitats are often phenotypically similar as larvae, and container dwelling subgenera are sometimes grouped together in diagnostic keys [11–14].

Although Belkin speculated that ground pool dwelling was likely ancestral in this group [15], the evolution of habitat specialization to container dwelling has remained largely unstudied and thus untested in the Aedini. This is likely due in part to the challenge of reconstructing the evolutionary history of the group itself, exemplified by the taxonomic confusion surrounding the Aedini. In recent years, nomenclature of *Aedes* mosquitoes has been repeatedly adjusted based on morphology alone; the genus of more than 900 species was divided into two genera by elevation of the subgenus *Ochlerotatus* to generic status (with several aedine subgenera placed within it) [16], followed by the additional elevation of more than 70 subgenera to generic status [17–19], all of which were returned to a single genus when subsequent analyses of the same morphological dataset failed to recover the same clades that caused the elevation of so many subgenera [6]. These nomenclature adjustments have received varying levels of support within the research community [6, 20, 21], but they highlight the degree of uncertainty that exists in relatedness of these mosquitoes. There has been a notable lack of densely sampled molecular phylogenies in this group; all have either been heavily restricted geographically and have used only a single marker [22, 23] or have contained only a handful of Aedini species [10, 24]. However, numerous studies have explored population level relationships within species of the Aedini [25–27], or utilized diagnostic regions for differential identification [28–31]; thus, a wealth of DNA sequences are available in digital repositories for this group.

Here we use comparative methods to evaluate evolutionary hypotheses regarding larval habitat specialization and larval morphology in the Aedini. We begin by building the most densely sampled phylogeny for this group to date, and use this phylogeny to explore the evolutionary processes underlying habitat specialization with stochastic character mapping and ancestral character state estimation under maximum likelihood, with the hypothesis that the ground pool larval habitat is ancestral. Next, we ask whether adaptation to these habitats might explain the phenotypic diversity we observe in larval characteristics. We quantify morphological variation with a multivariate approach using previously published morphological data, and then test the hypothesis that larval phenotypes are related based on habitat specialization, when accounting for phylogeny. We then compare different models of evolution, including the multivariate Ornstein-Uhleinbeck (OU) model, which accounts for different adaptive peaks in a macroevolutionary landscape to test whether the phenotypic diversity observed might result from adaptation to different larval habitats.

## Results

### Alignment and maximum likelihood phylogeny of the Aedini

We used nomenclature for genera following Wilkerson [6], but also indicated an alternative and previous nomenclature [17], primarily corresponding to subgenera, by indicating present subgenus. We generated sequence data for 81 species, which we augmented with public sequence data for an additional 179 species (Additional file 1: Table S2), across a total of seven markers (Additional file 1: Table S3). During alignment preparation, we excluded four aedine species which failed quality control steps as these single-marker species had nearest BLAST hits outside of their genera or subgenera and resolved outside of their genera or subgenera in preliminary phylogenetic analyses, despite all other members of those genera or subgenera being monophyletic (see Methods and Additional file 2: Supplemental Results). Our final alignment encompassed sequences from 260 species from all 10 genera in the Aedini, 222 of which were species in the genus *Aedes*, from 43 subgenera of *Aedes*. The alignment was a total length of 6298 bases, with an average coverage of 1940 nucleotides per taxa. We had depth of two markers or more for 167 taxa, and three markers or more for 104 taxa. This alignment was partitioned according to the best supported scheme from PartitionFinder 2 [32]; for all partitions, the best substitution model was GTR + G (Additional file 1: Table S4).

**Fig. 1:**
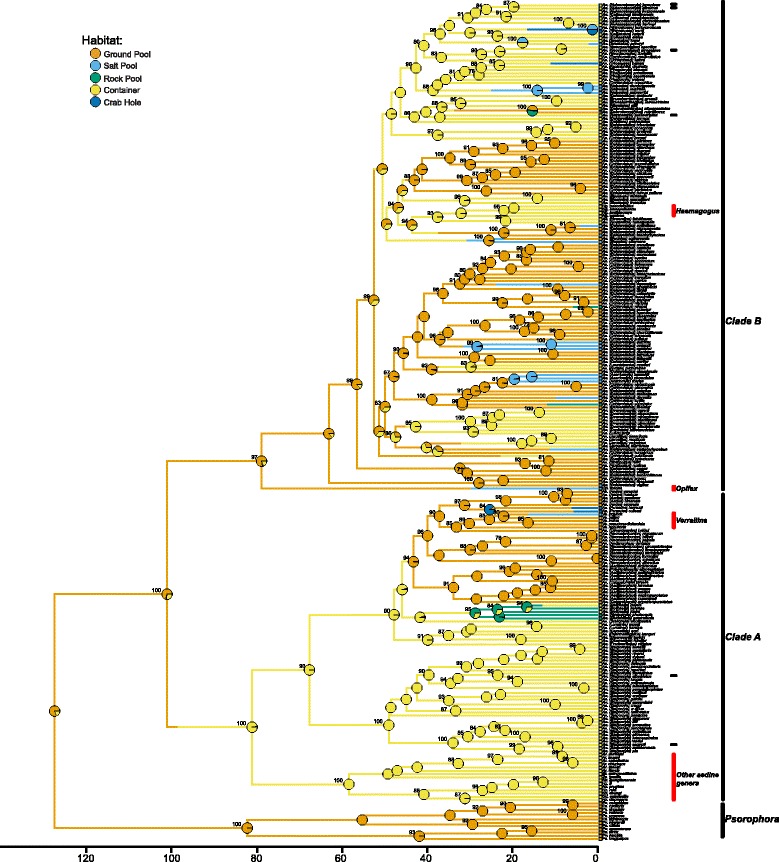
The maximum likelihood phylogeny from our analysis of the Aedini from seven markers, rendered ultrametric with chronos from the R package APE. Habitat transitions and putative ancestral character states from one of our stochastic character maps are presented. SH-like branch support values above 80 are shown as numbers on branches, while circles at nodes indicate posterior probabilities of a given habitat type. Scale is in millions of years. Horizontal bars near tips indicate invasive species, from top to bottom: *Ae. (Hulecoeteomyia) japonicus*, *Ae. (Hulecoeteomyia) koreicus, Ae. (Georgecraigius) atropalpus*, *Ae. (Rampamyia) notoscriptus*, *Ae. (Stegomyia) albopictus, and Ae. (Stegomyia) aegypti.* Boxes at tips indicate current habitat type; tips with multi-color boxes are taxa with more than one habitat specialization. *Aedes* is not monophyletic, invasive taxa are not monophyletic, and all non-*Psorophora* aedines fall into two large clades, here called Clade A and Clade B. Genera violating the monophyly of *Aedes* are shown with red labels. Bolded taxa are represented by more than one marker in our phylogenetic analysis

This multisequence alignment was used in RAxML version 8.2.9 [33] on CIPRES [34] to infer the maximum likelihood phylogeny. We assessed support on our final Maximum Likelihood tree (lnL = −87,380.17) using the non-parametric Shimodaira-Hasegawa-Like approximation of a likelihood ratio test [35], with a conservative threshold of 85 or higher to consider a clade supported [36, 37]. We found no relationship between length in alignment and terminal branch length (Kendall’s τ = −0.07, z = −1.67, *P* = 0.09), indicating that branch lengths in general were not biased due to missing data. As the penalized likelihood method we used to ultrametricize our phylogeny (see Methods, below) for comparative analyses does not output estimates of uncertainty around node ages, and because it is not directly informed by sequence variation, we focus our results here on evolutionary relationships between aedines, rather than on estimates of clade ages. Our maximum likelihood phylogeny recovered many strongly supported clades, particularly at nodes deep in the phylogeny and near the tips. We found that *Psorophora* was sister to all other aedines (Fig. 1, Additional file 3: Figure S1). We also found that that all other aedine taxa resolved in two well-supported clades: the first with a SH-like branch support value of 100 encompasses the majority of non-*Aedes* aedine genera of *Armigeres*, *Eretmapodites*, *Heizmannia*, and *Udaya*, as well as many *Aedes* subgenera such as *Stegomyia*, *Aedimorphus*, *Aedes*, and others, hereafter ‘Clade A’; and this clade is sister to a second clade with a SH-like branch support value of 98, hereafter ‘Clade B’, which includes the non-*Aedes* genera *Opifex* and *Haemagogus*, as well as the majority of *Aedes* subgenera such as *Ochlerotatus*, *Rampamyia*, *Hulecoeteomyia*, and many others (Fig. 1, Additional file 3: Figure S1). Both lineages A and B contain invasive species: within A are the notable disease vectors *Aedes (Stegomyia) albopictus* and *Aedes (Stegomyia) aegypti*, while in B, the invasive *Aedes (Rampamyia) notoscriptus*, *Aedes (Hulecoeteomyia) japonicus*, *Aedes (Hulecoeteomyia) koreicus*, and *Aedes (Georgecraigius) atropalpus*. Thus, *Aedes* was not monophyletic, and nor were invasive *Aedes* species; neither was the genus *Heizmannia*, although all taxa from the genus *Heizmannia* were represented exclusively from sequences on GenBank, as was the taxon that violated monophyly of this genus, *Udaya subsimilis*. All other aedine genera represented by more than one taxon were monophyletic. Additionally, 21 subgenera were recovered as monophyletic out of the 26 subgenera with more than one taxon in our analysis. *Aedimorphus*, *Catageoimyia*, and *Neomelaniconion* (in Clade A) and *Ochlerotatus*, *Phagomyia*, and *Collesius* (in Clade B) were not monophyletic, although in the case of *Neomelaniconion*, *Phagomyia* and *Collesius* monophyly was violated by taxa with only a single marker. We found the same qualitative relationships as those described above for our maximum likelihood analysis involving the 104 taxa with more than three markers (Additional file 4: Figure S2).

### Convergence of habitat specialization in the Aedini

We found strong evidence that the ground pool habitat was ancestral in the Aedini. Aedine mosquitoes utilize five different types of larval habitats, although the most common habitats are containers and ground pools (Table 1, Additional file 1: Table S1). We found a model with equal transition rates between habitats was favored by model weighting (Additional file 1: Table S5), and that both our stochastic character mapping and maximum likelihood-based ancestral state reconstruction yielded the strongest support for ground pools as ancestral for all *Aedes* taxa and the Aedini as a whole (Fig. 1, Additional file 5: Figure S3). As the results of both stochastic character mapping and the maximum likelihood-based ancestral state reconstruction were qualitatively the same, we present only the results of our stochastic character mapping here. The posterior probability for the ancestor of the Aedini utilizing ground pool habitats was 0.80, compared to a posterior probability of 0.18 for container habitats, and the posterior probability for the ancestor of *Aedes* utilizing ground pool habitats was 0.71 compared to a posterior probability of 0.28 for container habitats. Additionally, our results suggested that container dwelling first evolved on the branch leading to Clade A, while there were multiple transitions to container dwelling within Clade B. Thus, an ecological specialization in which the aquatic life stages utilize container habitats appears to have evolved multiple times within the Aedini, and on different branches leading to invasive species (Fig. 1). Salt pool and crab hole specialists arose from putatively ground pool lineages, save for two instances of each in Clade B, which arose from a putatively container dwelling lineage. Rock pool specialists arose from both container dwelling and ground hole dwelling lineages. The results from our stochastic character mapping involving taxa with only three or more markers and on the uncalibrated maximum likelihood topology provided the same qualitative results, but with even higher posterior probability and likelihood of a ground dwelling ancestor for the Aedini and for *Aedes* (see Additional file 2: Supplemental Results, Additional File 9: Figure S7, Additional File 11: Figure S9, Additional File 12: Figure S10).

### Convergence of morphology in the Aedini

**Fig. 2:**
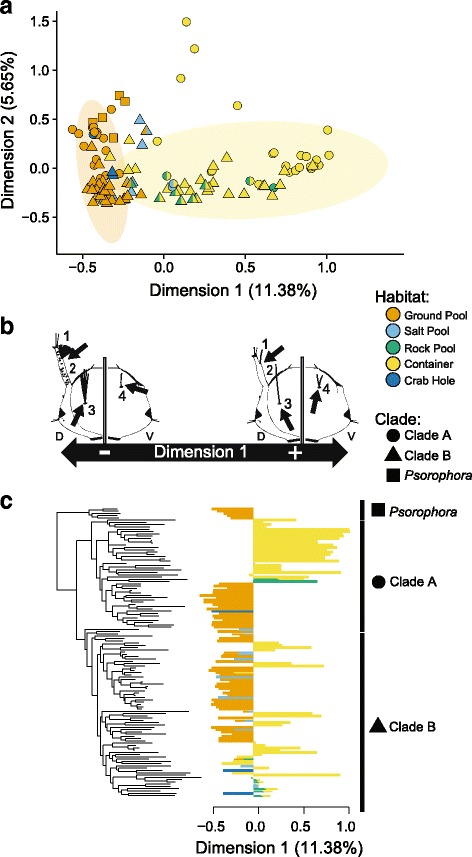
Evidence of convergence in the Aedini. Here, clade membership in plots is indicated with symbols, while colors indicate the habitat preference. **a** A biplot of dimensions 1 and 2 from our MCA for 127 Aedini, with a 95% confidence ellipse drawn around container (yellow) and ground pool dwelling (orange) taxa. Some container taxa appear more similar to one another, regardless of clade of origin, while ground pool taxa fall within a small range of negative values on dimension 1, regardless of clade of origin. **b** Several examples of convergent characters on the cranium of the mosquito contrasted between positive values on dimension one (typical of container mosquitoes) and negative values on dimension one (typical of ground pool mosquitoes). D stands for dorsal, and V for ventral. *1:* Seta 1A, either single/double branched, or with >3 branches. *2:* Antennae spicules either absent or present. *3:* Seta 5C, either single or multibranched. *4:* Seta 14C, either single or multi-branched. **c** The maximum likelihood phylogeny trimmed to include only those species with morphological data, with dimension 1 plotted alongside tips, colored according to larval habitat. Multiple lineages of Aedini have converged on strongly positive values in dimension 1, while other lineages maintain conserved negative values in dimension 2

Next, we evaluated whether larval morphology might reflect the convergence observed in habitat preference by converting categorical variables of larval morphology to continuous dimensions using a multiple correspondence analysis (MCA) and keeping the first five dimensions. Our MCA was based on 89 nominal morphological characters for 127 Aedines for which we had both molecular and morphological data. The first dimension of our MCA explained 11.38% of the variation in our larval character data set, while the second explained 5.65%; cumulatively, the five dimensions we kept for analysis explained more variation (28.1%) than the last 91 dimensions combined (27.3%). A biplot revealed that container dwelling mosquitoes occupied similar phenotypic space, regardless of clade membership, as did ground pool dwelling mosquitoes (Fig. 2a). Container species had, on average, higher scores on dimension one compared to ground pool mosquitoes and there were no ground pool mosquitoes with positive scores on dimension one. Both salt pool specialists and crab hole specialists clustered with ground pool mosquitoes, while rock pool mosquitoes clustered with container mosquitoes (Fig. 2a); in fact, all rock hole specialists fell within the 95% confidence ellipse for container mosquitoes, and all crab hole specialists fell within the ellipse for ground pool mosquitoes. All but seven salt marsh mosquitoes fell within the confidence ellipse for ground pool mosquitoes, and none had positive values on dimension one. Larger scores on the first dimension were generally but not exclusively associated with fewer branches of setae, for instance on the head capsule (Fig. 2b, Additional file 6: Figure S4), larval segments, and ventral brush, while lower values on dimension one were often associated with multiple branching patterns (Additional file 1: Table S6).

We found significant phylogenetic signal in all five dimensions used in our analysis; notably, dimensions one and two had Blomberg’s K values of 1.2 (*P* < 0.01) and 2.7 (P < 0.01) respectively, suggesting that in these dimensions, variation exceeded neutral expectations and was distributed among clades, rather than concentrated within clades (Table 2).

**Table 2:** Estimates of Blomberg’s k and w for the five morphological dimensions used in this analysis

Blomberg’s K			Wheathsheath Index (w)			
Dimension	k	P^a^	Container	P^a^	Ground Pool	P^a^
1	1.21	0.001	1.18	0.03	6.02	<0.001
2	2.78	0.001	0.97	0.52	1.07	0.37
3	0.84	0.001	0.76	0.97	1.83	<0.001
4	0.49	0.022	0.71	0.99	1.63	<0.001
5	1.03	0.001	0.91	0.65	1.61	<0.001

^a^P-values derived from 1000 simulations

We explicitly tested whether morphological scores were associated with habitat preference using a phylogenetic informed multivariate analysis of variance (MANOVA) with our five morphological dimensions as response variables and larval habitat as the explanatory variable. Given that ground pool dwelling was ancestral and that container dwelling was convergent, a significant association of phenotype and habitat when accounting for phylogeny could indicate convergence of this phenotype. We expanded on existing methods for phylogenetic MANOVAs [38, 39] by generating null distributions for simulations using several additional models of evolution (see supplemental material for MANOVA code). We found that larval morphological scores were significantly associated with habitat specializations when accounting for phylogeny under models of Brownian Motion (BM), a purely neutral model via a ‘random walk’ [40]; Early Burst (EB), in which rates of Brownian motion decelerate along the phylogeny towards the tips [41]; and an Ornstein-Uhleinbeck (OU) process [40], in which a random walk is pulled towards one (OU1) distinct optimum (*P* < 0.001 for all MANOVA tests; see Additional file 1: Table S7). Moreover, separate lineages of container-dwelling *Aedes* mosquitoes exhibited positive scores on dimension 1, while ground pool sister clades of these lineages had negative scores on dimension one (Fig. 2c). Thus, container mosquitoes showed strong evidence of convergence in morphology, as they were more phenotypically similar to one another than the putatively ancestral ground pool mosquitoes and multiple lineages of container dwelling mosquitoes converged on these positive scores on dimension one.

Next, we used the Wheatsheaf index [42] to evaluate each dimension independently for the relative strength of convergence. The Wheatsheaf index (*w*) provides a relative measure of phenotypic similarity of a focal group and differentiation of this focal group from non-focus taxa, when accounting for phylogenetic distance; significant values of *w* in putatively convergent taxa suggest strong convergence, with relative values of *w* indicating the overall similarity of taxa within the convergent focal group and dissimilarity of this group from non-focal species. We found that the putatively convergent container mosquitoes were more similar to one another than expected due to phylogeny alone, thus showing strong convergence, but only for dimension one (Table 2, Additional file 7: Figure S5). Notably, ground pool mosquitoes showed a remarkably similar phenotype, particularly on dimension one (Fig. 2a, Fig. 2c, Additional file 7: Figure S5).

### Adaptive evolution explains Aedine morphology

We then used a model selection approach to evaluate the hypothesis that the morphological convergence we observed could be explained by adaptive evolution. We compared models that approximated different evolutionary processes acting on our morphological dimensions: Brownian Motion, Early Burst, and Ornstein-Uhleinbeck processes. For OU models, we used a model with a single optimum (‘OU1’) that represented an adaptive landscape with a single optimum regardless of habitat type, and the three best scoring models with different optima per habitat type and habitat shifts represented by stochastic character mapping (OUM A, B, and C), representing an adaptive landscape with different peaks for each habitat type. As we hypothesized, OU processes with different adaptive peaks for each habitat specialization were the best fitting models (best scoring model AICc = −226.12, cumulative AICc Weight of OUM models = 0.99; Table 3; full model parameters given in Additional file 1: Table S8; the three best scoring stochastic character maps are given in Additional File 8: Figure S6). Moreover, standard deviations of simulated mean values of dimension one from the best scoring model overlapped with real values of dimension one for all taxa (Additional file 10: Figure S8). When the best scoring model is used as a null model for a phylogenetic MANOVA with habitat specialization as the explanatory variable and our observed five dimensions as response variables, this null model is a plausible distribution for our data (*P* = 0.42; see Additional file 1: Table S7).

**Table 3:** A model comparison of the fit of different models of evolution to our five dimensions of morphological trait data

Model	AICCc	ΔAICC	Weight
OUM1	−234.38	0	0.99
OUM2	−221.69	12.68	1.75E-03
OUM3	−221.12	13.25	1.3E-03
EB	−204.12	30.26	2.68E-07
BM	−166.46	67.92	1.78E-15
OU1	−126.90	107.48	4.56e-24

## Discussion

Use of container larval habitats has been key to the success and global dispersal of *Aedes* mosquito lineages, as these mosquitoes have been transported principally through global trade. *Aedes (Stegomyia) aegypti* is thought to have dispersed throughout tropical and sub-tropical regions of the world during the slave trade and subsequent colonial mercantile activities several hundred years ago [43], while *Aedes (Stegomyia) albopictus* and other invasive *Aedes* mosquitoes such as *Aedes (Hulecoeteomyia) japonicus* have spread more recently due to the international trade in used tires and lucky bamboo plants [2, 3, 7, 8]. These and other invasive mosquitoes from multiple lineages have exploited human activity for dispersal, but it is only because of the repeated colonization of container habitats by aedine lineages that *Aedes* mosquitoes have become the global disease vectors and pests they are today, especially in densely populated urban environments. Moreover, our models suggest that this convergence in habitat specialization has been accompanied by convergent adaptation to container habitats, explaining the similarity in larval phenotype between distant aedine relatives that we have quantified here, and that others have noted, such as the less robust ventral brush among container dwelling mosquitoes [11].

The phenotypic convergence we observed is not complete across all taxa. While container habitat specialists trended towards positive values on dimension one, not all container specialists had positive values and container habitat specialists were more variable than ground pool breeders (Fig. 2, Additional file 7: Figure S5). There are three potential reasons for this. First, lineages in Clade B evolved container dwelling larvae after those in Clade A, and there may have been insufficient time for lineages to have converged on the same phenotypic values in all Clade B lineages, an explanation consistent with simulations predicting lower scores for container dwelling mosquitoes in Clade B relative to Clade A (Additional file 10: Figure S8). Second, our method of categorizing habitat specialization, although consistent with the literature [15, 17], fails to capture the diversity of habitats utilized by many of these mosquitoes (Table 1) and thus may fail to capture potential differences in selective pressures dependent on specific differences within habitat types, such as the difference between a rotting tree hole and a coconut shell. Finally, we may have incompletely sampled convergent characters, leading to incomplete resolution of a convergent phenotype in our MCA (that is, our characters used in this analysis are unlikely to represent all convergent characters in these taxa). Regardless of the reason for variation along dimension one, our OU models provide a plausible explanation for the variation observed in morphological dimensions, as true trait values along dimension one fell within two standard deviations of model-simulated traits in all cases.

In contrast to container dwelling mosquitoes, ground pool species exhibited remarkably conserved phenotypes across all three major clades identified in this study (Fig. 2 A, Additional file 7: Figure S5). These results are all the more remarkable considering the ancient divergence time between these clades (*Psorophora* and Clades A and B) on the order of 100 MYA [10]. This suggests to us that ground pool mosquitoes represent an ancient ancestral phenotype related to habitat specialization and larval morphology, and further that the ancestral aedine mosquito utilized ground pool habitats and had a negative phenotype on dimension one (Figs. 1 and 2). If our analyses are incorrect regarding the ancestral habitat specialization of the Aedini, and ground pool mosquitoes are a convergent phenotype (i.e. container dwelling is ancestral), then we have alternatively detected convergence in these taxa. However, barring this possibility, our results suggest likely adaptive processes driving niche conservatism and thus constraining phenotype in ground pool mosquitoes. Moreover, the striking difference between related ground pool and container mosquitoes highlight how selection can act within clades in markedly different ways, depending on ecological specialization.

Due to the relatively small number of species available in our analysis utilizing salt pool, crab hole, and rock pool habitats, we primarily discuss and contrast the evolution of container and ground pool specialization. None the less, it is worth highlighting the similarity in phenotype between these less common habitat specializations and the more common ground pool and container mosquitoes. For instance, salt pool and crab hole specialists occupy the same phenotypic space as ground pool specialists (Fig. 2), and arise primarily from putative ground pool lineages (Fig. 1). The same is true for rock pool specialists and container specialists; rock pool specialists almost always occupy the same phenotypic space as container specialists. Thus, at least for the species in our analysis, salt pool and crab hole specialists exhibit a phenotype we associate with the ancestral ground pool mosquitoes, while rock pool specialists exhibit a convergent phenotype, similar to container mosquitoes. These results may explain why some subgenera of mosquitoes contain species which utilize both salt pool and ground pool habitats, and why many rock pool mosquitoes may also occupy container habitats (Fig. 1, Table 1, Additional file 1: Table S1).

Often, convergence occurs due to adaptation to similar niches in separate geographic regions [44–46], although convergence may also occur in sympatric species rich communities, where the number of species exceeds the number of available niches [47, 48]. It seems likely that the majority of strong convergence between lineages of clades A and B occurred in geographic isolation. The majority of taxa in Clade A are found in the Afrotropical and Oriental regions [17], while taxa from Clade B are found in Australia, the Nearctic, Neotropical, and Palearctic regions [17] (Additional file 1: Table S5). Lineages that are most similar phenotypically along dimension one are isolated between continents. For example, the subgenus *Aedes (Stegomyia)* in Clade A is found natively in Africa, Asia, and some Pacific islands [17], while the phenotypically similar lineage leading to *Aedes (Howardina)* and *Haemagogus* in Clade B is found exclusively in the Neotropics and parts of the southern Nearctic [17]. However, many of the container occupying lineages in Clade B, such as *Collesius* and *Hulecoeteomyia*, have overlapping ranges with lineages in Clade A in eastern and southeastern Asia [17]. Interestingly, these lineages in Clade B have lower scores on dimension one, raising the possibility that despite frequently sharing habitat types (and even occasionally being found in the same habitats), these taxa may be occupying different microhabitats that are contributing to a different phenotype.

Although we have detected convergence and plausibly attributed it to adaptive processes, it is difficult to identify what direct benefit the component morphological structures of our dimensions (primarily presence, absence, or robustness of setae) might confer to mosquito larvae in one habitat versus another. These structures could relate either directly or indirectly to traits that influence foraging or predator avoidance. Container habitats are generally smaller and more confining than ground pools, and habitat size has been negatively correlated with aquatic predator abundance [49, 50]. However, it is thought that larval behavior is the predominant form of antipredator defense in *Aedes* mosquitoes [51] and the functional role of setal structures as it may relate to predator defense is unknown. Alternatively, as we cannot identify the function of the phenotypic variation we identified, there remains a possibility that larval morphology itself is not adaptive to larval habitats, but rather, it is linked to other phenotypes that are adaptive.

Our study adds to the existing literature finding that *Aedes* is not monophyletic [10, 17, 24], and our finding of at least two major clades containing *Aedes* mosquitoes concurs with previous molecular phylogenies [22, 24]. Moreover, our results are largely consistent with morphological observations on sexual anatomy of groups of subgenera [16] that elevated *Ochlerotatus* to generic status and placed all of the subgenera in our Clade B within that genus, a change which was later reversed [6]. Our results are also similar to quantitative cladistic analyses that delineated *Aedes* into at least two non-monophyletic clades [17–19], although we recovered different relationships within clades (See Supplemental for additional discussion on phylogenetic relationships recovered). Because our sampling relied heavily on GenBank sequences, and often on taxa with incomplete coverage, we would caution against the use of our phylogeny for the purposes of explicit taxonomic action. Rather, our results highlight the increasing need for a densely sampled molecular phylogeny of aedine mosquitoes, preferably drawing from vouchered samples exclusively, and not relying on a public database. Given the public health importance of these disease vectors and the vital role of a consistent nomenclature in communication among scientists, future efforts to resolve the taxonomy of this group should rely on more complete sampling both in the number of taxa and marker coverage. However, our phylogeny casts significant doubt on the appropriateness of a single genus *Aedes* for all 929 species currently classified as such.

## Conclusion

In summary, we have shown that the use of container habitats, a key component of the invasive success of several medically important mosquito species, has resulted from convergent evolution from a ground pool dwelling ancestor, with multiple aedine lineages converging on container habitat specialization. Moreover, we have demonstrated that variation in larval phenotypes may be associated with adaptation to selective pressures in different larval habitat types, as we detected strong evidence of convergence of phenotype in container mosquitoes, and significant conservation of phenotype in ground pool mosquitoes. Further, our results highlight the need for further study in this important mosquito group, as we provide substantial evidence that the medically important genus *Aedes* is not monophyletic.

## Methods

### Mosquito samples

We collected and solicited mosquito specimens from researchers and mosquito control workers across six continents. DNA was isolated from one specimen per mosquito species following manufacturers protocols with the EZNA Forensic DNA Extraction kit (Omega Biotek, Norcross, GA), after which it was stored at −20 °C. Mosquito specimen information is presented in Additional file 1: Table S2. All remaining DNA from samples is stored at the Connecticut Agricultural Experiment Station.

### Sequencing and GenBank data acquisition

Partial sequences were amplified from the small subunit ribosomal RNA (18S), the large subunit ribosomal RNA (28S), cytochrome oxidase subunit 1 (COI) [52], the internal transcribed spacer 2 region (ITS2) [23], arginine kinase [10], and enolase [10], using either published primers or those we designed (Additional file 1: Table S2). All PCRs were conducted in reaction volumes of 25 μl containing 12.5 μl of GoTaq Green Master Mix (Promega, Madison, WI), 1 μl of each primer, up to 3 μl of DNA extraction. Thermal cycling conditions were as follows: 94 °C for 5 min, then 35 cycles of 94 °C for 30s, 50 °C for 1 m, 72 °C for 1 m, followed by a final extension of 72 °C for 5 min. PCR products were directly sequenced in both directions by Macrogen (Macrogen Boston USA, Cambridge, MA), then assembled in Geneious [53]. Our sequencing data was augmented with sequences from a GenBank pipeline in the R [54] package megaptera (available from the author at https://github.com/heibl/megaptera) for all aforementioned markers, plus cytochrome oxidase subunit II. The package megaptera queries GenBank and retrieves sequences from taxa of interest (set in this case to the Aedini), compares them to a reference, generates species-level consensus sequences, and provides them as output alignments for all markers of interest. All sequences from this pipeline were validated with BLAST and with preliminary phylogenetic analyses. Additional details on megaptera, our quality control procedure, and our query terms (Additional file 1: Table S2) are provided in the supplemental materials. In total, the seven markers used represented all markers for which there were more than 30 different aedine species sequences on GenBank at the time of this study. All sequences for *Culex quinquefasciatus*, our outgroup, were retrieved from GenBank.

### Alignment and phylogenetic analyses

Data from our sequencing were combined with the GenBank pipeline, and used MAFFT [55] in Geneious to realign all sequences, with G-INS-I settings for protein coding genes, and E-INS-I settings for ribosomal RNA genes and ITS2. For ITS2, we also aligned sequences manually as ITS2 can be difficult to align due to its variability. All alignments were concatenated, and used PartitionFinder 2 [32] to find the best partition scheme and substitution model given a base of partitions with each marker separate, and each protein coding gene also partitioned by codon position. Our concatenated alignment was then used in RAxML version 8.2.9 [33] on CIPRES [34] with the best fitting partition scheme from PartitionFinder 2. Maximum likelihood phylogenies were estimated from 100 distinct starting runs on randomized maximum parsimony trees (flag -N 100). The resulting phylogeny was passed back to RAxML, where Shimodaira-Hasegawa-like (SH-like) approximations of likelihood ratio tests were performed across the tree (flag -f J). SH-like branch supports have a general null hypothesis that a “branch is incorrect,” and compare a branch to sub-optimal Nearest Neighbor Interchange rearrangements [35]. SH-like branch supports have several advantages over other support metrics: generating these values is substantially faster [36, 56], may provide more accurate values for short branches when compared with bootstrap values [37, 57], and are robust to matrices with missing data [35]. We also generated a maximum likelihood phylogeny with SH-like branch support for taxa for which there were three or more markers to ensure that our results were not biased due to samples without high marker coverage.

Our phylogeny was visualized in FigTree (available at http://tree.bio.ed.ac.uk/software/figtree/) and TreeGraph2 [58]. We rerooted the phylogeny along the branch leading to *Cx. quinquefasciatus* and evaluated if missing data biased our branch length estimates by testing for a correlation with Kendall’s τ between terminal branch lengths and proportion of nucleotides in the alignment. We considered clades to be well supported following a conservative threshold of SH-like branch support values at 85 or higher, indicating an 85% chance that a branch was ‘correct’ [36, 37].

### Ancestral state reconstruction

We used previously published larval habitat information [17, 59, 60] and other publicly available resources (The Mosquito Taxonomic Inventory available at http://mosquito-taxonomic-inventory.info) to assign a larval habitat to all but one of our taxa, *Aedes (Ochlerotatus) euiris*, whose larval habitat we were unable to determine. Many comparative analyses require an ultrametric tree, such as those available from divergence time analyses. However, there is only one aedine fossil that can be reliably placed relative to extant genera, and while other mosquito fossils can be reliably placed relative to other extant genera, at this time we lack the taxonomic coverage in our molecular data to complete an extensive divergence time analysis incorporating such fossils. Thus, we chose instead to ultrametricize our maximum likelihood phylogenies using penalized likelihood [61, 62] in chronos from the R package ape with the default correlated rates model [63]. We provided chronos with three calibration points. The first two calibration points were drawn from Reidenbach et al.’s fossil-calibrated Bayesian relaxed clock analysis [10]: the common ancestor of *Culex* and *Aedes* between 226.22 and 172.28 MYA, a timing that is consistent with recent molecular clock analyses from whole genome data [64]; and the common ancestor of all aedine mosquitoes between 155.71 and 90.18 MYA. The final calibration point was based on the only aedine fossil placed in an extant genus or subgenus, *Ochlerotatus serafini* [65], an Eocene fossil found in Baltic amber dating to between 33.9 and 55.8 MYA. This final fossil-informed calibration was placed as the common ancestor of all *Aedes (Ochlerotatus)* species, with a minimum age of 33.9 MYA and a maximum age of 80.26 MYA, the extent of the 95% confidence interval on the age estimate of divergence between *Aedes* (*Ochlerotatus) triseriatus* and *Haemagogus equinus* in Reidenbach et al. [10]. For all comparative analyses, we trimmed *Cx. quinquefasciatus* from the phylogeny to avoid the outgroup biasing our results. Then, we performed our ancestral state reconstruction on three phylogenies: the time-calibrated maximum likelihood phylogeny of all species for which we had habitat data, the time-calibrated phylogeny from species represented by three or more markers, and the maximum likelihood phylogeny, as some authors have argued that calibrating/ultrametricizing a tree may lose important branch length information [66]. We then used stochastic character mapping to evaluate the ancestral habitat of the Aedini, providing priors for all taxa based on present habitat specialization, with an equal probability for a habitat for taxa occupying more than one habitat. We used AIC weights to evaluate three transition rate models: equal rates, symmetrical transition rates (where the rates are the same transitioning to or from one specific habitat to another specific habitat), or a model where all rates could vary between transitions to and from larval habitats. We generated 1000 stochastic character maps with make.simmap from the R package phytools with the best fitting transition model [67]. These maps were summarized with posterior probabilities at nodes in order to infer the best supported ancestral state for a given node. We repeated this character mapping, as above, on our phylogeny from three or more markers and used likelihood-based ancestral character state estimation for discrete characters with the function rayDISC from the R package corHMM [68].

### Convergence of larval morphology

We characterized variation in larval phenotype with a multiple correspondence analysis on a published categorical character matrix of larval morphology [17] using the MCA function in the R package factomineR [69]. Ordered characters were excluded, as the MCA function does not presently differentiate between ordinal and nominal categorical variables. This MCA generated uncorrelated numeric dimensions that described the primary trends in phenotypic variation among our mosquito species that we used in subsequent analyses as indicators of phenotypic similarity between species. For all comparative analyses, we used time-calibrated maximum likelihood phylogenies, described above. We estimated phylogenetic signal with Blomberg’s K [70] using phylosig from phytools [67]. To test whether larval phenotype across habitats was more similar than expected due to the phylogeny alone, we implemented a phylogenetically informed MANOVA following aov.phylo in the R package Geiger [38, 39], but which allowed for null distributions to be simulated under EB, OU1, or a model specified by the user, in addition to Brownian Motion, utilizing the package the R mvMORPH. Habitat preference was used as the explanatory variable, and all five larval dimensions as the response variables. Next, we used the Wheatsheaf index [42] to evaluate the strength of convergence of phenotype across each dimension with the R package windex [71], specifying focal groups from which to measure phenotypic similarity as container mosquitoes and ground pool mosquitoes. The Wheatsheaf index (w) provides a relative measure of a focal group’s similarity to other focal members, and their relative difference from non-focal species, while correcting for expected phylogenetic similarity under neutral expectations (Brownian motion). Focal groups must be defined a priori and with knowledge of the process that generated their similarity (e.g. convergent evolution), as the measure cannot distinguish why a focal group exceeds neutral expectations, such as might be the case in either convergence or conservation of phenotype [42]. As we have relatively few taxa for habitat specialists other than ground pool or container specialists, we considered only comparisons between container and ground pool specialists with the Wheatsheaf index.

#### Model comparisons

Finally, we evaluated the hypothesis that adaptation to larval habitats might plausibly explain the variation along morphological dimensions we observed by comparing several different multivariate models of evolution implemented in the package mvMORPH [72]. Models of evolution were fit for Browning Motion, so called ‘random walk’; Early Burst, where rates of Brownian Motion decay with time; and Ornstein-Uhleinbeck processes, in which change in trait values occurs toward, and is constrained by, one or several optima that are analogous to adaptive peaks (50). We used both single-optimum OU processes (OU1, mimicking natural selection along a phylogeny without any shifts in optima) and multi-optima OU processes (OUM, wherein there was a different optimum for each habitat specialization, and shifts in optima occurred with shifts in habitat preference). We fit each of these modes to our phylogenetic tree, trimmed to taxa for which we had morphological scores, and our morphological dimensions with default parameters, save for OU models, where we disabled estimation of the root state as per the author’s recommendations for phylogenies containing only extant taxa [72]. To choose plausible scenarios of optima shifts along our phylogeny for our OUM models, we took a random sample of 100 stochastic character maps of larval habitat, trimmed each map to taxa for which we had morphological data, and estimated a model for each, using the three best scoring OUM models in our subsequent model comparison. AICs were then used to compare model weights with the function aicw in geiger. We validated that our best scoring model was a good approximation for our data by comparing its simulations with our real data, both as the null distribution in our phylogenetic MANOVA, and by comparing our real data to the standard deviation of mean simulated values from 1000 simulations of the OUM model with the base R function simulate [54].

## Additional files

Additional file 1: s_tablesv1. Figure Captions and Tables. This document provides supplemental **Tables S1-S7.**, as well as supplemental figure captions. **Table S8.** contains model results from the evolutionary models compared in Soghigian et al. (DOCX 56 kb)

Additional file 2: supplement_textv1. Supplemental methods, results, and discussion. This document provides additional details on our methods, as well as consideration of our phylogenetic results and a discussion of their significance in this supplemental material. (DOCX 22 kb)

Additional file 3: **Figure S1.** The maximum likelihood phylogeny from RAxML with all taxa included. Genera that violate the monophyly of Aedes are highlighted in red. The tree has been rooted leading to the branch *for Culex quinquefasciatus*. Scale is in substitutions per site. Calibration points used in this study have been indicated with numbered arrows. Details on calibration points are given in our methods. 1: The common ancestor of *Culex* and *Aedes* between 226.22 and 172.28 MYA. 2: The common ancestor of all aedine mosquitoes between 155.71 and 90.18 MYA. 3: The common ancestor of all *Aedes (Ochlerotatus)* species, with a minimum age of 33.9 MYA and a maximum age of 94.29 MYA. (PDF 401 kb)

Additional file 4: **Figure S2.** The maximum likelihood phylogeny from an alignment containing only taxa with three or more markers. Scale is in substitutions per site. We still recover Clade A and B from our full analysis in this analysis, containing 104 taxa with high marker coverage. Genera that violate the monophyly of Aedes are highlighted in red. (PDF 217 kb)

Additional file 5: **Figure S3.** Maximum likelihood ancestral character reconstruction of discrete characters suggests that ground pool dwelling was ancestral in the Aedini and in Aedes. The time-calibrated maximum likelihood phylogeny from our analysis of the Aedini showing putative ancestral character states from our maximum likelihood analysis of discrete characters is shown here. Size of pie slices shows the likelihood of a habitat type. (PDF 1.27 mb)

Additional file 6: **Figure S4.** A comparison of four larval mounts, two from container dwelling mosquitoes, *Aedes (Ochlerotatus) triseriatus and Aedes (Stegomyia) albopictus* and two from ground pool dwelling mosquitoes *Aedes (Ochlerotatus) excrucians* and *Aedes (Aedimorphus) vexans*. *Aedes (Stegomyia) albopictus* and *Aedes (Aedimorphus) vexans* are in Clade A, *while Aedes (Ochlerotatus) triseriatus and Aedes (Ochlerotatus) excrucians* are in Clade B. (PDF 8.19 mb)

Additional file 7: **Figure S5.** The time-calibrated maximum likelihood phylogeny from our analysis of the Aedini showing habitat transitions and putative ancestral character states from one of our stochastic character maps, along with the five dimensions from our multiple correspondence analysis, colored by habitat preference. (PDF 673 kb)

Additional file 8: **Figure S6.** The three stochastic character maps representing the best-scoring OUM models 1, 2, and 3 from Table 3. Full model parameters are given in Table S8. (PDF 517 kb)

Additional file 9: **Figure S7.** One stochastic character map from our Bayesian stochastic character map of our reduced data set containing 103 taxa for which we had marker coverage of three or more. As in the case of our complete analysis, we find that ground pool dwelling was likely ancestral in both the Aedini as a whole and in the genus *Aedes*. (PDF 41 kb)

Additional file 10: **Figure S8.** Mean simulated values along dimension 1 (black dots) with two standard deviations (whiskers). Real values on dimension 1 shown as colored circles. Real values fall within two standard deviations of all simulated values. (PDF 307 kb)

Additional file 11: **Figure S9.** One stochastic character map from our stochastic character map on our untransformed maximum likelihood phylogeny. As in the case of our complete analysis, we find that ground pool dwelling was likely ancestral in both the Aedini as a whole and in the genus *Aedes*. Size of pie slices shows the posterior probability of a habitat type. (PDF 609 kb)

Additional file 12: **Figure S10.** The maximum likelihood ancestral character reconstruction of discrete characters along our untransformed maximum likelihood phylogeny suggests that ground pool dwelling was ancestral in the Aedini and in *Aedes*. Size of pie slices shows the likelihood of a habitat type. (PDF 1.25 mb)

Additional file 13: soghigian_et_al_alignment_parts.txt. Partition scheme for the nucleotide alignment. The best partition scheme was determined using PartitionFinder 2, and is listed here. (TXT 389 bytes)

Additional file 14: soghigian_et_al_ml.nwk. Newick file for the maximum likelihood phylogeny of the Aedini. The maximum likelihood phylogeny generated in this study presented in newick format. (NWK 18 kb)

Additional file 15: soghigian_et_al_calibrated.nwk. Newick file for the calibrated phylogeny of the Aedini. The maximum likelihood phylogeny of the Aedini was rendered ultrametric and calibrated for usage in comparative analyses, and presented here in newick format. (NWK 14 kb)

Additional file 16: soghigian_et_al_ml_3ormore.nwk. Newick file for the maximum likelihood phylogeny of the Aedini generated from taxa with three or more markers. The maximum likelihood phylogeny generated in this study for taxa with three or more markers, representing taxa for which we have high confidence in sequence identity, in newick format. (NWK 7 kb)

Additional file 17: soghigian_et_al_alignment.phy. The alignment used in maximum likelihood analyses. The alignment used in maximum likelihood analyses from more than 200 aedines in phylip format. (PHY 1606 kb)

Additional file 18: phylo_anova.R. Phylogenetic ANOVA and MANOVA Code. This is the phylogenetic ANOVA/MANOVA code we used to test for relationships between dimensions and habitat specializations under different evolutionary models. Has not been extensively tested with other datasets. See code for details. (R 4 kb)

Additional file 19: supplemental_data.xlsx. Habitat Specializations and Morphological Dimensions of the Aedini. This spreadsheet contains the habitat specializations used in this analysis and the first five morphological dimensions analyzed in the manuscript. (XLSX 27 kb)

## Abbreviations

AICc: Small-sample-size corrected Akaike information criterion
BM: Brownian motion
EB: Early murst
MANOVA: Multivariate analysis of variance
MYA: Million years ago
OU: Ornstein-Uhleinbeck model
OU1: Single optima OU model
OUM: Multi-Optima Ornstein-Uhleinbeck model
SH-like: Shimodaira-Hasegawa-Like approximation of a likelihood ratio test
