# Supplementary material for: From ground pools to treeholes: convergent evolution of habitat and phenotype in Aedes mosquitoes
Source: BMC Evol Biol. 2017 Dec 19;17:262. doi: 10.1186/s12862-017-1092-y (PMC5735545; doi:10.1186/s12862-017-1092-y)
Supplement: Supplementary file 1 — s_tablesv1. Figure Captions and Tables. This document provides supplemental Tables S1-S7., as well as supplemental figure captions. Table S8. contains model results from the evolutionary models compared in Soghigian et al. (DOCX 56 kb) [file 12862_2017_1092_MOESM1_ESM.docx]

SUPPLEMENTAL TABLES

| Supplemental Table 1. The genera of the Aedini, *Aedes* subgenera*, and clade in this analysis (for taxa present), as well as the total species of the subgenus, the species in the present analysis, the larval habitat (if known), and the bioregion in which the genus/subgenus can be found. For clade in this analysis, A refers to Clade A, Clade B, or *Psorophora* (See Figure 1). For bioregion, we follow Reinert et al. 2009 (19): 1, Nearctic; 2, Palaearctic; 3, Afrotropical; 4, Oriental; 5, Australasian and western Pacific Islands; 6, Neotropical. Subgenera with currently invasive taxa are represented by parentheses around a region. | | | | | | | |
| --- | --- | --- | --- | --- | --- | --- | --- |
| Genus | Genus sensu Reinert 2000 | Subgenus, genus sensu Reinert et al. 2009 or earlier | Clade in analysis | Total species* | Species in analysis | Larval Habitat* | Region Found* |
| *Aedes (Ae.)* | *Ochlerotatus* | *Abraedes* |  | 1 |  | Container | 1 |
| *Aedes (Ae.)* | *Ochlerotatus* | *Acartomyia* | B | 3 | 3 | Salt Pools | 2 |
| *Aedes (Ae.)* | *Aedes* | *Aedes* | A | 12 | 5 | Ground Pool | 2 |
| *Aedes (Ae.)* | *Aedes* | *Aedimorphus* | A | 66 | 10 | Ground Pool | 1,2,3,4,5 |
| *Aedes (Ae.)* | *Aedes* | *Alanstonea* |  | 2 |  | Container | 4 |
| *Aedes (Ae.)* | *Aedes* | *Albuginosus* |  | 9 |  | Container | 3 |
| *Aedes (Ae.)* | *Aedes* | *Ayurakitia* |  | 2 |  | Continaer | 4 |
| *Aedes (Ae.)* | *Ochlerotatus* | *Aztecaedes* |  | 1 |  | Container, Rock Pool | 1 |
| *Aedes (Ae.)* | *Aedes* | *Belkinius* |  | 1 |  | Container | 4 |
| *Aedes (Ae.)* | *Aedes* | *Bifidistylus* |  | 4 |  | Rock Pool | 3 |
| *Aedes (Ae.)* | *-* | *Borichinda* | A | 1 | 1 | Rock Pool | 4 |
| *Aedes (Ae.)* | *Aedes* | *Bothaella* | A | 6 | 3 | Rock Pool, Container | 4 |
| *Aedes (Ae.)* | *Ochlerotatus* | *Bruceharrisonius* | B | 9 | 2 | Container | 2 |
| *Aedes (Ae.)* | *Aedes* | *Cancraedes* |  | 10 |  | Crab Hole, Salt Pool | 4 |
| *Aedes (Ae.)* | *Aedes* | *Catageiomyia* | A | 28 | 3 | Ground Pool | 3 |
| *Aedes (Ae.)* | *Aedes* | *Catatassomyia* |  | 1 |  | Container | 4 |
| *Aedes (Ae.)* | *Aedes* | *Christophersiomyia* |  | 5 |  | Container | 4 |
| *Aedes (Ae.)* | *Ochlerotatus* | *Collesius* | B | 9 | 4 | Rock Pool | 4 |
| *Aedes (Ae.)* | *Aedes* | *Cornetius* | A | 1 | 1 | Container | 3 |
| *Aedes (Ae.)* | *Ochlerotatus* | *Dahliana* | B | 3 | 1 | Container | 2 |
| *Aedes (Ae.)* | *Ochlerotatus* | *Danliesa* | B | 3 | 1 | Container | 4 |
| *Aedes (Ae.)* | *Aedes* | *Dendroskusea* |  | 5 |  | Container | 4 |
| *Aedes (Ae.)* | *Aedes* | *Diceromyia* | A | 14 | 2 | Container | 3,4 |
| *Aedes (Ae.)* | *Ochlerotatus* | *Dobrotworskyius* | B | 7 | 2 | Rock Pool, Ground Pool | 5 |
| *Aedes (Ae.)* | *Ochlerotatus* | *Downsiomyia* | B | 30 | 6 | Container | 4 |
| *Aedes (Ae.)* | *Aedes* | *Edwardsaedes* | A | 3 | 1 | Ground Pool | 2,4,5 |
| *Aedes (Ae.)* | *Aedes* | *Elpeytonius* |  | 2 |  | Container | 3 |
| *Aedes (Ae.)* | *Ochlerotatus* | *Finlaya* |  | 36 |  | Container | 4,5 |
| *Aedes (Ae.)* | *Aedes* | *Fredwardsius* | A | 1 | 1 | Rock Pool, Container | 2,3,4 |
| *Aedes (Ae.)* | *Ochlerotatus* | *Georgecraigius* | B | 3 | 3 | Rock Pool, Container | 1,(2),6 |
| *Aedes (Ae.)* | *Aedes* | *Geoskusea* | A | 10 | 1 | Crab Hole, Salt Pool | 4 |
| *Aedes (Ae.)* | *Ochlerotatus* | *Gilesius* |  | 2 |  | Rock Pool, Container | 2,4 |
| *Aedes (Ae.)* | *Ochlerotatus* | *Gymnometopa* | B | 1 | 1 | Container | 6 |
| *Aedes (Ae.)* | *Ochlerotatus* | *Halaedes* | B | 3 | 3 | Salt Pool | 5 |
| *Aedes (Ae.)* | *Ochlerotatus* | *Himalaius* | B | 2 | 1 | Container | 4 |
| *Aedes (Ae.)* | *Ochlerotatus* | *Hopkinsius* |  | 7 |  | Container | 3 |
| *Aedes (Ae.)* | *Ochlerotatus* | *Howardina* | B | 34 | 2 | Container | 6 |
| *Aedes (Ae.)* | *Aedes* | *Huaedes* |  | 3 |  | Container | 5 |
| *Aedes (Ae.)* | *Ochlerotatus* | *Hulecoeteomyia* | B | 14 | 5 | Rock Pool, Container | 4 |
| *Aedes (Ae.)* | *Aedes* | *Indusius* |  | 1 |  | Unknown | 4 |
| *Aedes (Ae.)* | *Aedes* | *Isoaedes* |  | 1 |  | Rock Pool | 4 |
| *Aedes (Ae.)* | *Ochlerotatus* | *Jarnellius* | B | 5 | 4 | Container | 1 |
| *Aedes (Ae.)* | *Ochlerotatus* | *Jihlienius* |  | 3 |  | Container | 4 |
| *Aedes (Ae.)* | *Ochlerotatus* | *Kenknightia* | B | 12 | 1 | Container | 4 |
| *Aedes (Ae.)* | *Ochlerotatus* | *Kompia* | B | 1 | 1 | Container | 1 |
| *Aedes (Ae.)* | *Aedes* | *Leptosomatomyia* |  | 1 |  | Container | 5 |
| *Aedes (Ae.)* | *Aedes* | *Levua* |  | 1 |  | Crab Hole | 5 |
| *Aedes (Ae.)* | *Ochlerotatus* | *Lewnielsenius* | B | 1 | 1 | Container | 1 |
| *Aedes (Ae.)* | *Aedes* | *Lorrainea* | A | 5 | 2 | Container | 4,5 |
| *Aedes (Ae.)* | *Ochlerotatus* | *Luius* | B | 1 | 1 | Container | 4 |
| *Aedes (Ae.)* | *Ochlerotatus* | *Macleaya* | B | 11 | 3 | Container | 5 |
| *Aedes (Ae.)* | *Ochlerotatus* | *Molpemyia* |  | 3 |  | Container | 5 |
| *Aedes (Ae.)* | *Ochlerotatus* | *Mucidus* | B | 14 | 2 | Ground Pool, Salt Pool | 4,5 |
| *Aedes (Ae.)* | *Aedes* | *Neomelaniconion* | A | 28 | 9 | Ground Pool | 3,4,5 |
| *Aedes (Ae.)* | *-* | *Nyctomyia* | A | 2 | 1 | Rock Pool | 4 |
| *Aedes (Ae.)* | *Ochlerotatus* | *Ochlerotatus* | B | 258 | 86 | All | 1,2,3,4,5,6 |
| *Aedes (Ae.)* | *Aedes* | *Paraedes* | A | 8 | 2 | Crab Hole | 4 |
| *Aedes (Ae.)* | *Ochlerotatus* | *Patmarksia* |  | 13 |  | Container | 5 |
| *Aedes (Ae.)* | *Aedes* | *Petermattinglyius* | A | 5 | 1 | Container | 4 |
| *Aedes (Ae.)* | *Ochlerotatus* | *Phagomyia* | B | 16 | 4 | Container, Rock Pool | 4 |
| *Aedes (Ae.)* | *Aedes* | *Polyleptiomyia* |  | 2 |  | Ground Pool | 3 |
| *Aedes (Ae.)* | *Aedes* | *Pseudarmigeres* |  | 5 |  | Container | 3 |
| *Aedes (Ae.)* | *Ochlerotatus* | *Rampamyia* | B | 3 | 1 | Container | (1),5 |
| *Aedes (Ae.)* | *Ochlerotatus* | *Rhinoskusea* | B | 4 | 2 | Crab Hole | 4,5 |
| *Aedes (Ae.)* | *Ochlerotatus* | *Rusticoidus* | B | 10 | 3 | Ground Pool | 1,2 |
| *Aedes (Ae.)* | *Ochlerotatus* | *Sallumia* |  | 2 |  | Ground Pool, Crab Hole | 6 |
| *Aedes (Ae.)* | *Aedes* | *Scutomyia* | A | 9 | 1 | Container | 4,5 |
| *Aedes (Ae.)* | *Aedes* | *Skusea* |  | 4 |  | Crab Hole | 3 |
| *Aedes (Ae.)* | *Aedes* | *Stegomyia* | A | 128 | 33 | Container | (1),2,3,4,5,(6) |
| *Aedes (Ae.)* | *Ochlerotatus* | *Tanakaius* | B | 2 | 2 | Salt Pool, Container | 1,2,4 |
| *Aedes (Ae.)* | *Aedes* | *Tewarius* |  | 3 |  | Container | 4 |
| *Aedes (Ae.)* | *Ochlerotatus* | *Vansomerenis* |  | 3 |  | Container | 3 |
| *Aedes (Ae.)* | *Ochlerotatus* | *Zavortinkius* |  | 11 |  | Container | 4 |
| *Armigeres (Ar.)* | *-* | *-* | A | 58 | 6 | Container | 2,4,5 |
| *Eretmapodites (Er.)* | *-* | *-* | A | 48 | 1 | Container | 3 |
| *Haemagogus (Hg.)* | *-* | *-* | B | 28 | 4 | Container | 6 |
| *Heizmannia (Hz.)* | *-* | *-* | A | 38 | 6 | Container | 4 |
| *Opifex (Op.)* | *-* | *-* | B | 2 | 1 | Salt Pool | 5 |
| *Psorophora (Ps.)* | *-* | *-* | P | 50 | 12 | Ground Pool | 1,6 |
| *Udaya (Ud.)* | *-* | *-* | A | 3 | 1 | Container | 4 |
| *Verrallina (Ve.)* | *-* | *-* | A | 95 | 5 | Ground Pool | 2,4,5 |
| *Zeugnomyia* | *-* | *-* | A | 4 | 1 | Container | 4 |
| *Total species, larval habitat information, and mosquito distribution data primarily drawn from Reinert et al. 2009 (19), but also augmented from the Mosquito Taxonomic Inventory (http://mosquito-taxonomic-inventory.info), where necessary. | | | | | | | |

| Supplemental Table 2. Summary table for taxa in this analysis. We include habitat and sequence coverage for all taxa used in this analysis. Sequences that originated from our GenBank pipeline are indicated by "GBP." Continent of origin and accession numbers are provided for species we sequenced. | | | | | | | | | |
| --- | --- | --- | --- | --- | --- | --- | --- | --- | --- |
| Taxa | Habitat* | 18S | 28S | Arginine Kinase | COI | COII | Enolase | ITS2 | Collection Region |
| *Ae. (Acartomyia) mariae* | Salt pool |  |  |  | GBP |  |  |  |  |
| *Ae. (Acartomyia) phoeniciae* | Salt pool |  |  |  | GBP |  |  | GBP |  |
| *Ae. (Acartomyia) zammitii* | Salt pool |  |  |  | GBP |  |  | GBP |  |
| *Ae. (Aedes) cinereus* | Ground pool | MG232543 | MG242558 | MG232443 | MG242481 |  | MG232475 | MG232614 | MA, USA |
| *Ae. (Aedes) esoensis* | Ground pool |  | GBP |  | GBP |  |  |  |  |
| *Ae. (Aedes) geminus* | Ground pool |  |  |  |  |  |  | GBP |  |
| *Ae. (Aedes) rossicus* | Ground pool |  |  |  | GBP |  |  |  |  |
| *Ae. (Aedes) yamadai* | Ground pool |  |  |  | GBP |  |  |  |  |
| *Ae. (Aedimorphus) centropunctatus* | Ground pool | MG232541 | MG242556 |  | MG242479 |  |  |  | SEN |
| *Ae. (Aedimorphus) cumminsii* | Ground pool | MG232546 | MG242561 | MG232440 | MG242484 |  | MG232477 |  | SEN |
| *Ae. (Aedimorphus) dalzieli* | Ground pool | MG232547 | MG242562 | MG232439 | MG242485 |  | MG232478 |  | SEN |
| *Ae. (Aedimorphus) dentatus* | Ground pool |  |  |  | GBP |  |  |  |  |
| *Ae. (Aedimorphus) fowleri* | Ground pool | MG232555 | MG242570 | MG232432 | MG242493 |  | MG232485 |  | SEN |
| *Ae. (Aedimorphus) hirsutus* | Ground pool | MG232561 | MG242576 | MG232429 | MG242499 |  | MG232490 |  | SEN |
| *Ae. (Aedimorphus) mediolineatus* | Ground pool |  | GBP |  | GBP | GBP |  |  |  |
| *Ae. (Aedimorphus) ochraceus* | Ground pool |  |  |  | GBP |  |  | GBP |  |
| *Ae. (Aedimorphus) pallidostriatus* | Ground pool |  |  |  | GBP |  |  | GBP |  |
| *Ae. (Aedimorphus) vexans* | Ground pool | MG232586 | MG242602 | MG232406 | MG242525 | GBP | MG232513 | MG232641 | MA, USA |
| *Ae. (Borichinda) cavernicola* | Rock pool |  |  |  | GBP |  |  | GBP |  |
| *Ae. (Bothaella) helenae* | Rock pool |  |  |  | GBP | GBP |  |  |  |
| *Ae. (Bothaella) kleini* | Rock pool |  |  |  | GBP | GBP |  |  |  |
| *Ae. (Bothaella) manhi* | Container |  |  |  | GBP | GBP |  |  |  |
| *Ae. (Bruceharrisonius) aureostriatus* | Container |  |  |  | GBP | GBP |  |  |  |
| *Ae. (Bruceharrisonius) greenii* | Container |  | GBP |  | GBP |  |  |  |  |
| *Ae. (Catageiomyia) argenteopunctatus* | Ground pool | MG232530 | MG242545 | MG232453 | MG242468 |  | MG232464 |  | SEN |
| *Ae. (Catageiomyia) tarsalis* | Ground pool |  |  |  | GBP |  |  |  |  |
| *Ae. (Collessius) elsiae* | Rock pool, container |  | GBP |  | GBP | GBP |  |  |  |
| *Ae. (Collessius) hatorii* | Rock pool, container |  | GBP |  | GBP |  |  |  |  |
| *Ae. (Collessius) pseudotaeniatus* | Rock pool, container |  |  |  | GBP |  |  |  |  |
| *Ae. (Collessius) tonkinensis* | Rock pool, container |  | GBP |  | GBP | GBP |  |  |  |
| *Ae. (Cornetius) cozi**(60) | Container | MG232545 | MG242560 | MG232441 | MG242483 |  | MG232476 |  | SEN |
| *Ae. (Dahliana) geniculatus* | Container | MG232558 | MG242573 | MG232430 | MG242496 |  | MG232488 | MG232621 | HUN |
| *Ae. (Danielsia) albotaeniatus* | Container |  | GBP |  | GBP | GBP |  |  |  |
| *Ae. (Diceromyia) furcifer* | Container | MG232557 | MG242572 | MG232431 | MG242495 | GBP | MG232487 |  | SEN |
| *Ae. (Diceromyia) taylori* | Container | MG232580 | MG242596 | MG232411 | MG242519 | GBP | MG232508 |  | SEN |
| *Ae. (Dobrotworskyius) alboannulatus* | Ground pool, rock pool | MG232527 | MG242542 | MG232455 | MG242465 |  | MG232462 | MG232602 | AUS |
| *Ae. (Dobrotworskyius) rubrithorax* | Ground pool, rock pool |  |  |  | GBP |  |  | GBP |  |
| *Ae. (Downsiomyia) albolateralis* | Container |  | GBP |  | GBP | GBP |  |  |  |
| *Ae. (Downsiomyia) nipponicus* | Container |  | GBP |  | GBP |  |  | GBP |  |
| *Ae. (Downsiomyia) nishikawai* | Container |  |  |  | GBP |  |  |  |  |
| *Ae. (Downsiomyia) niveoides* | Container |  | GBP |  | GBP | GBP |  |  |  |
| *Ae. (Downsiomyia) novoniveus* | Container |  | GBP |  | GBP | GBP |  |  |  |
| *Ae. (Downsiomyia) omorii* | Container |  | GBP |  | GBP | GBP |  |  |  |
| *Ae. (Edwardsaedes) bekkui* | Ground pool |  |  |  | GBP |  |  |  |  |
| *Ae. (Fredwardsius) vittatus* | Rock pool, container | MG232588 | MG242604 | MG232404 | MG242527 | GBP | MG232515 |  | SEN |
| *Ae. (Georgecragius) fluviatilis* | Rock pool, container |  |  |  | GBP |  |  |  |  |
| *Ae. (Georgecraigius) atropalpus* | Rock pool, container | MG232532 | MG242547 | MG232451 | MG242470 | GBP | MG232466 | MG232605 | MA, USA |
| *Ae. (Georgecraigius) epactius* | Rock pool, container | MG232552 | MG242567 | MG232434 | MG242490 |  | MG232482 |  | TX, USA |
| *Ae. (Geoskusea) baisasi* | Crab hole |  |  |  | GBP |  |  |  |  |
| *Ae. (Gymnometopa) mediovittatus* | Container |  |  |  | GBP |  |  |  |  |
| *Ae. (Halaedes) ashworthi* | Salt pool |  |  |  |  | GBP |  | GBP |  |
| *Ae. (Halaedes) australis* | Salt pool |  |  |  |  | GBP |  | GBP |  |
| *Ae. (Halaedes) wardangensis* | Salt pool |  |  |  |  | GBP |  | GBP |  |
| *Ae. (Himalaius) gilli* | Container |  | GBP |  | GBP | GBP |  |  |  |
| *Ae. (Howardina) bahamensis* | Container |  |  |  | GBP |  |  | GBP |  |
| *Ae. (Howardina) fulvithorax* | Container |  |  |  | GBP |  |  | GBP |  |
| *Ae. (Hulecoeteomyia) chrysolineatus* | Rock pool, container |  | GBP |  | GBP | GBP |  |  |  |
| *Ae. (Hulecoeteomyia) formosensis* | Container |  | GBP |  | GBP | GBP |  |  |  |
| *Ae. (Hulecoeteomyia) harveyi* | Container |  |  |  | GBP | GBP |  |  |  |
| *Ae. (Hulecoeteomyia) japonicus* | Rock pool, container | MG232565 | MG242580 | MG232425 | MG242503 | GBP | MG232494 | MG232625 | MA, USA |
| *Ae. (Hulecoeteomyia) koreicus* | Rock pool, container |  | GBP |  | GBP |  |  | GBP |  |
| *Ae. (Jarnellius) deserticola* | Container |  |  |  | GBP |  |  |  |  |
| *Ae. (Jarnellius) monticola* | Container |  |  |  | GBP |  |  |  |  |
| *Ae. (Jarnellius) sierrensis* | Container | MG232574 | MG242590 | MG232416 | MG242513 |  | MG232503 | MG232634 | UT, USA |
| *Ae. (Jarnellius) varipalpus* | Container |  |  |  | GBP |  |  |  |  |
| *Ae. (Kenknightia) dissimilis* | Container |  |  |  | GBP |  |  |  |  |
| *Ae. (Kompia) purpureipes* | Container |  |  |  | GBP |  |  |  |  |
| *Ae. (Lewnielsenius) muelleri* | Container |  |  |  | GBP |  |  |  |  |
| *Ae. (Lorrainea) amesii* | Container |  |  |  | GBP |  |  |  |  |
| *Ae. (Lorrainea) fumidus* | Container |  |  |  | GBP |  |  |  |  |
| *Ae. (Luius) fengi* | Container |  |  |  | GBP |  |  |  |  |
| *Ae. (Macleaya) macmillani* | Container |  |  |  | GBP |  |  | GBP |  |
| *Ae. (Macleaya) tremulus* | Container |  |  |  | GBP |  |  | GBP |  |
| *Ae. (Macleaya) wattensis* | Container |  |  |  | GBP |  |  | GBP |  |
| *Ae. (Mucidus) alternans* | Ground pool, salt pool |  |  |  | GBP |  |  | GBP |  |
| *Ae. (Mucidus) sudanensis* | Ground pool | MG232578 | MG242594 |  | MG242517 |  |  |  | SEN |
| *Ae. (Neomelaniconion) albiradius* | Ground pool |  |  |  |  |  |  | GBP |  |
| *Ae. (Neomelaniconion) belleci* | Ground pool |  |  |  |  |  |  | GBP |  |
| *Ae. (Neomelaniconion) circumluteolus* | Ground pool |  |  |  | GBP |  |  | GBP |  |
| *Ae. (Neomelaniconion) fontenillei* | Ground pool |  |  |  |  |  |  | GBP |  |
| *Ae. (Neomelaniconion) lineatopennis* | Ground pool |  |  |  | GBP | GBP |  |  |  |
| *Ae. (Neomelaniconion) mcintoshi* | Ground pool |  |  |  | GBP |  |  | GBP |  |
| *Ae. (Neomelaniconion) nigropterum* | Ground pool |  |  |  |  |  |  | GBP |  |
| *Ae. (Neomelaniconion) palpale* | Ground pool |  |  |  |  |  |  | GBP |  |
| *Ae. (Neomelaniconion) sylvaticum* | Ground pool |  |  |  |  |  |  | GBP |  |
| *Ae. (Nyctomyia) pholeocola* | Ground pool |  |  |  | GBP |  |  | GBP |  |
| *Ae. (Ochlerotatus) abserratus* | Rock pool | MG232524 | MG242539 | MG232458 | MG242462 |  | MG232459 | MG232600 | MA, USA |
| *Ae. (Ochlerotatus) albifasciatus* | Ground pool | GBP |  |  | GBP |  |  |  |  |
| *Ae. (Ochlerotatus) aloponotum* | Ground pool |  |  |  | GBP |  |  |  |  |
| *Ae. (Ochlerotatus) angustivittatus* | Ground pool |  |  |  | GBP |  |  |  |  |
| *Ae. (Ochlerotatus) annulipes* | Ground pool | MG232529 | MG242544 |  | MG242467 |  |  |  |  |
| *Ae. (Ochlerotatus) atlanticus* | Ground pool | MG232531 | MG242546 | MG232452 | MG242469 |  | MG232465 | MG232604 | FL, USA |
| *Ae. (Ochlerotatus) aurifer* | Ground pool | MG232533 | MG242548 | MG232450 | MG242471 |  | MG232467 | MG232606 | PA, USA |
| *Ae. (Ochlerotatus) bancroftianus* | Ground pool | MG232534 | MG242549 | MG232449 | MG242472 |  | MG232468 | MG232607 | AUS |
| *Ae. (Ochlerotatus) behningi* | Ground pool |  |  |  | GBP |  |  |  |  |
| *Ae. (Ochlerotatus) bicristatus* | Ground pool |  |  |  | GBP |  |  |  |  |
| *Ae. (Ochlerotatus) bimaculatus* | Ground pool | GBP |  |  | GBP |  |  |  |  |
| *Ae. (Ochlerotatus) brelandi* | Ground pool | MG232535 | MG242550 | MG232448 | MG242473 |  | MG232469 | MG232608 | TX, USA |
| *Ae. (Ochlerotatus) burgeri* | Container |  |  |  | GBP |  |  |  |  |
| *Ae. (Ochlerotatus) campestris* | Container |  |  |  | GBP |  |  |  |  |
| *Ae. (Ochlerotatus) camptorhynchus* | Ground pool | MG232537 | MG242552 | MG232446 | MG242475 |  | MG232471 | MG232610 | AUS |
| *Ae. (Ochlerotatus) canadensis* | Salt pool | MG232538 | MG242553 |  | MG242476 |  | MG232472 |  | MA, USA |
| *Ae. (Ochlerotatus) cantans* | Ground pool |  |  |  | GBP |  |  |  |  |
| *Ae. (Ochlerotatus) cantator* | Ground pool | MG232539 | MG242554 | MG232445 | MG242477 |  |  | MG232611 | MA, USA |
| *Ae. (Ochlerotatus) caspius* | Salt pool | MG232540 | MG242555 | MG232444 | MG242478 | GBP | MG232473 | MG232612 | GRC |
| *Ae. (Ochlerotatus) cataphylla* | Ground pool, salt pool | GBP |  |  | GBP |  |  |  |  |
| *Ae. (Ochlerotatus) churchillensis* | Ground pool | MG232542 | MG242557 |  | MG242480 |  | MG232474 | MG232613 | CO, USA |
| *Ae. (Ochlerotatus) coluzzii* | Ground pool |  | GBP |  |  |  |  | GBP |  |
| *Ae. (Ochlerotatus) communis* | Salt pool | MG232544 | MG242559 | MG232442 | MG242482 |  |  | MG232615 | MA, USA |
| *Ae. (Ochlerotatus) crinifer* | Ground pool | GBP |  |  | GBP |  |  |  |  |
| *Ae. (Ochlerotatus) cyprius* | Ground pool |  |  |  | GBP |  |  |  |  |
| *Ae. (Ochlerotatus) detritus* | Ground pool | MG232548 | MG242563 | MG232438 | MG242486 |  | MG232479 | MG232616 | GRC |
| *Ae. (Ochlerotatus) diantaeus* | Salt pool | MG232549 | MG242564 | MG232437 | MG242487 |  |  | MG232617 | ON, CAN |
| *Ae. (Ochlerotatus) dorsalis* | Ground pool | MG232550 | MG242565 | MG232436 | MG242488 | GBP | MG232480 | MG232618 | PA, USA |
| *Ae. (Ochlerotatus) dupreei* | Ground pool | MG232551 | MG242566 | MG232435 | MG242489 |  | MG232481 |  | FL, USA |
| *Ae. (Ochlerotatus) eatoni* | Salt pool |  |  |  | GBP |  |  |  |  |
| *Ae. (Ochlerotatus) euedes* | Container |  |  |  | GBP |  |  |  |  |
| *Ae. (Ochlerotatus) euiris* | Ground pool |  |  |  | GBP |  |  |  |  |
| *Ae. (Ochlerotatus) excrucians* | Ground pool | MG232553 | MG242568 |  | MG242491 |  | MG232483 | MG232619 | MA, USA |
| *Ae. (Ochlerotatus) fitchii* | Ground pool | MG232554 | MG242569 | MG232433 | MG242492 |  | MG232484 | MG232620 | PA, USA |
| *Ae. (Ochlerotatus) flavescens* | Ground pool |  |  |  | GBP |  |  |  |  |
| *Ae. (Ochlerotatus) fulvus pallens* | Ground pool | MG232556 | MG242571 |  | MG242494 |  | MG232486 |  | TX, USA |
| *Ae. (Ochlerotatus) grossbecki* | Ground pool | MG232559 | MG242574 |  | MG242497 |  |  |  |  |
| *Ae. (Ochlerotatus) hendersoni* | Container | MG232560 | MG242575 |  | MG242498 |  | MG232489 | MG232622 | MA, USA |
| *Ae. (Ochlerotatus) hexodontus* | Ground pool |  | GBP |  | GBP |  |  | GBP |  |
| *Ae. (Ochlerotatus) hungaricus* | Ground pool |  |  |  | GBP |  |  |  |  |
| *Ae. (Ochlerotatus) impiger* | Ground pool |  |  |  | GBP |  |  | GBP |  |
| *Ae. (Ochlerotatus) implicatus* | Ground pool | MG232562 | MG242577 | MG232428 | MG242500 |  | MG232491 | MG232623 | CO, USA |
| *Ae. (Ochlerotatus) increpitus* | Ground pool |  |  |  | GBP |  |  |  |  |
| *Ae. (Ochlerotatus) infirmatus* | Ground pool | MG232563 | MG242578 | MG232427 | MG242501 |  | MG232492 |  | FL, USA |
| *Ae. (Ochlerotatus) intrudens* | Ground pool | MG232564 | MG242579 | MG232426 | MG242502 |  | MG232493 | MG232624 | PA, USA |
| *Ae. (Ochlerotatus) kasachstanicus* | Ground pool |  | GBP |  | GBP | GBP |  |  |  |
| *Ae. (Ochlerotatus) leucomelas* | Ground pool |  |  |  | GBP |  |  |  |  |
| *Ae. (Ochlerotatus) mallochi* | Container |  |  |  | GBP |  |  | GBP |  |
| *Ae. (Ochlerotatus) melanimon* | Ground pool |  | GBP |  | GBP |  |  |  |  |
| *Ae. (Ochlerotatus) mitchellae* | Ground pool | MG232569 | MG242584 | MG232421 | MG242507 |  | MG232498 |  | LA, USA |
| *Ae. (Ochlerotatus) nigrinus* | Ground pool |  |  |  | GBP |  |  |  |  |
| *Ae. (Ochlerotatus) nigripes* | Ground pool |  |  |  | GBP |  |  |  |  |
| *Ae. (Ochlerotatus) nigromaculis* | Ground pool |  | GBP |  | GBP |  |  |  |  |
| *Ae. (Ochlerotatus) niphadopsis* | Ground pool |  |  |  | GBP |  |  |  |  |
| *Ae. (Ochlerotatus) obturbator* | Rock pool |  |  |  | GBP |  |  |  |  |
| *Ae. (Ochlerotatus) oreophilus* | Container |  |  |  | GBP |  |  |  |  |
| *Ae. (Ochlerotatus) pertinax**(59) | Ground Pool | MG232571 | MG242586 | MG232419 | MG242509 |  | MG232500 | MG232630 | FL, USA |
| *Ae. (Ochlerotatus) pionips* | Ground pool |  |  |  | GBP |  |  | GBP |  |
| *Ae. (Ochlerotatus) procax* | Ground pool |  |  |  | GBP |  |  |  |  |
| *Ae. (Rusticoidus) provocans* | Ground pool |  | MG242587 |  | MG242510 |  |  | MG232631 | PA, USA |
| *Ae. (Ochlerotatus) pulcritarsis* | Container |  |  |  | GBP |  |  |  |  |
| *Ae. (Ochlerotatus) pullatus* | Ground pool |  |  |  | GBP |  |  |  |  |
| *Ae. (Ochlerotatus) punctor* | Ground pool | MG232572 | MG242588 | MG232418 | MG242511 |  | MG232501 | MG232632 | CT, USA |
| *Ae. (Rusticoidus) refiki* | Ground pool |  |  |  | GBP |  |  |  |  |
| *Ae. (Ochlerotatus) rempeli* | Rock pool |  |  |  | GBP |  |  |  |  |
| *Ae. (Ochlerotatus) riparius* | Ground pool |  |  |  | GBP |  |  |  |  |
| *Ae. (Rusticoidus) rusticus* | Ground pool |  |  |  | GBP |  |  |  |  |
| *Ae. (Ochlerotatus) sagax* | Ground pool | MG232573 | MG242589 | MG232417 | MG242512 |  | MG232502 | MG232633 | AUS |
| *Ae. (Ochlerotatus) scapularis* | Ground pool |  |  |  | GBP |  |  | GBP |  |
| *Ae. (Ochlerotatus) schizopinax* | Ground pool |  |  |  | GBP |  |  |  |  |
| *Ae. (Ochlerotatus) sollicitans* | Salt pool | MG232575 | MG242591 | MG232415 | MG242514 |  | MG232504 |  | CT, USA |
| *Ae. (Ochlerotatus) spencerii* | Ground pool |  |  |  | GBP |  |  | GBP |  |
| *Ae. (Ochlerotatus) squamiger* | Salt pool |  | GBP |  | GBP |  |  |  |  |
| *Ae. (Ochlerotatus) sticticus* | Ground pool | MG232576 | MG242592 | MG232414 | MG242515 |  | MG232505 | MG232635 | MA, USA |
| *Ae. (Ochlerotatus) stimulans* | Ground pool | MG232577 | MG242593 | MG232413 | MG242516 |  | MG232506 |  | CT, USA |
| *Ae. (Ochlerotatus) taeniorhynchus* | Ground pool, salt pool | MG232579 | MG242595 | MG232412 | MG242518 | GBP | MG232507 | MG232636 | MA, USA |
| *Ae. (Ochlerotatus) tahoensis* | Ground pool |  | GBP |  | GBP |  |  |  |  |
| *Ae. (Ochlerotatus) terrens* | Container |  |  |  | GBP |  |  | GBP |  |
| *Ae. (Ochlerotatus) thelcter* | Ground pool | MG232581 | MG242597 |  | MG242520 |  |  |  | TX, USA |
| *Ae. (Ochlerotatus) theobaldi* | Ground pool | MG232582 | MG242598 | MG232410 | MG242521 |  | MG232509 | GBP | AUS |
| *Ae. (Ochlerotatus) thibaulti* | Ground pool, container | MG232583 | MG242599 | MG232409 | MG242522 |  | MG232510 | MG232638 | PA, USA |
| *Ae. (Ochlerotatus) tormentor* | Ground pool |  |  |  |  |  |  | GBP |  |
| *Ae. (Ochlerotatus) tortilis* | Ground pool |  |  |  | GBP |  |  |  |  |
| *Ae. (Ochlerotatus) triseriatus* | Container | MG232584 | MG242600 | MG232408 | MG242523 | GBP | MG232511 | MG232639 | MA, USA |
| *Ae. (Ochlerotatus) trivittatus* | Ground pool | MG232585 | MG242601 | MG232407 | MG242524 |  | MG232512 | MG232640 | VT, USA |
| *Ae. (Ochlerotatus) ventrovittis* | Ground pool |  |  |  | GBP |  |  |  |  |
| *Ae. (Ochlerotatus) vigilax* | Ground pool, salt pool | MG232587 | MG242603 | MG232405 | MG242526 | GBP | MG232514 |  | AUS |
| *Ae. (Ochlerotatus) vittiger* | Ground pool | MG232589 | MG242605 |  | MG242528 |  |  | GBP | AUS |
| *Ae. (Ochlerotatus) zoosophus* | Container |  |  |  | GBP |  |  |  |  |
| *Ae. (Paraedes) barraudi* | Crab hole |  |  |  | GBP |  |  |  |  |
| *Ae. (Paraedes) collessi* | Crab hole |  |  |  | GBP |  |  |  |  |
| *Ae. (Petermattinglyius) iyengari* | Container |  |  |  | GBP |  |  |  |  |
| *Ae. (Phagomyia) assamensis* | Container |  | GBP |  | GBP | GBP |  |  |  |
| *Ae. (Phagomyia) khazani* | Container |  |  |  | GBP | GBP |  |  |  |
| *Ae. (Phagomyia) prominens* | Container |  | GBP |  | GBP | GBP |  |  |  |
| *Ae. (Phagomyia) watasei* | Container |  |  |  | GBP |  |  |  |  |
| *Ae. (Polyleptiomyia) albocephalus* | Ground pool |  |  |  |  |  |  | GBP |  |
| *Ae. (Rampamyia) notoscriptus* | Container | MG232570 | MG242585 | MG232420 | MG242508 | GBP | MG232499 | GBP | AUS |
| *Ae. (Rhinoskusea) portonovoensis* | Salt pool |  |  |  | GBP |  |  |  |  |
| *Ae. (Rhinoskusea) wardi* | Crab hole |  |  |  | GBP |  |  |  |  |
| *Ae. (Scutomyia) albolineatus* | Container |  |  |  | GBP | GBP |  |  |  |
| *Ae. (Stegomyia) aegypti* | Container | MG232525 | MG242540 | MG232457 | MG242463 | GBP | MG232460 | MG232601 | TX, USA |
| *Ae. (Stegomyia) africanus* | Container | MG232526 | MG242541 | MG232456 | MG242464 |  | MG232461 |  | SEN |
| *Ae. (Stegomyia) albopictus* | Container | MG232528 | MG242543 | MG232454 | MG242466 | GBP | MG232463 | MG232603 | BDA |
| *Ae. (Stegomyia) annandalei* | Container |  | GBP |  | GBP | GBP |  |  |  |
| *Ae. (Stegomyia) bromeliae* | Container | MG232536 | MG242551 | MG232447 | MG242474 |  | MG232470 | MG232609 | KEN |
| *Ae. (Stegomyia) craggi* | Container |  |  |  | GBP |  |  |  |  |
| *Ae. (Stegomyia) cretinus* | Container |  |  |  | GBP |  |  | GBP |  |
| *Ae. (Stegomyia) daitensis* | Container |  |  |  | GBP |  |  |  |  |
| *Ae. (Stegomyia) denderensis* | Container |  |  |  | GBP |  |  |  |  |
| *Ae. (Stegomyia) desmotes* | Container |  | GBP |  |  |  |  |  |  |
| *Ae. (Stegomyia) dybasi* | Container |  | GBP |  | GBP |  |  |  |  |
| *Ae. (Stegomyia) flavopictus* | Container |  | GBP |  | GBP |  |  | GBP |  |
| *Ae. (Stegomyia) galloisi* | Container |  | GBP |  | GBP |  |  |  |  |
| *Ae. (Stegomyia) hensilli* | Container |  | GBP |  | GBP |  |  |  |  |
| *Ae. (Stegomyia) lilii* | Container |  |  |  | GBP |  |  | GBP |  |
| *Ae. (Stegomyia) luteocephalus* | Container | MG232566 | MG242581 | MG232424 | MG242504 | GBP | MG232495 | MG232626 | SEN |
| *Ae. (Stegomyia) maehleri* | Container |  | GBP |  | GBP |  |  |  |  |
| *Ae. (Stegomyia) malikuli* | Container |  |  |  | GBP | GBP |  |  |  |
| *Ae. (Stegomyia) mascarensis* | Container | MG232567 | MG242582 | MG232423 | MG242505 |  | MG232496 | MG232627 | MUS |
| *Ae. (Stegomyia) metallicus* | Container | MG232568 | MG242583 | MG232422 | MG242506 |  | MG232497 | MG232628 | SEN |
| *Ae. (Stegomyia) palauensis* | Container |  | GBP |  | GBP |  |  |  |  |
| *Ae. (Stegomyia) pia* | Container |  |  |  | GBP |  |  | GBP |  |
| *Ae. (Stegomyia) polynesiensis* | Container |  |  |  |  |  |  | GBP |  |
| *Ae. (Stegomyia) pseudalbopictus* | Container |  | GBP |  | GBP | GBP |  |  |  |
| *Ae. (Stegomyia) pseudoscutellaris* | Container |  |  |  |  |  |  | GBP |  |
| *Ae. (Stegomyia) riversi* | Container |  | GBP |  | GBP |  |  | GBP |  |
| *Ae. (Stegomyia) scutellaris* | Container |  | GBP |  | GBP |  |  |  |  |
| *Ae. (Stegomyia) simpsoni* | Container |  |  |  | GBP |  |  | GBP |  |
| *Ae. (Stegomyia) subalbopictus* | Container |  | GBP |  | GBP | GBP |  |  |  |
| *Ae. (Stegomyia) tongae* | Container |  |  |  |  |  |  | GBP |  |
| *Ae. (Stegomyia) unilineatus* | Container |  |  |  | GBP |  |  |  |  |
| *Ae. (Stegomyia) w albus* | Container |  |  |  | GBP |  |  |  |  |
| *Ae. (Stegomyia) wadai* | Container |  | GBP |  | GBP |  |  |  |  |
| *Ae. (Tanakaius) savoryi* | Salt pool |  | GBP |  | GBP |  | GBP |  |  |
| *Ae. (Tanakaius) togoi* | Salt pool, container |  | GBP |  | GBP |  | GBP | GBP |  |
| *Ar. durhami* | Container |  | GBP |  | GBP |  |  |  |  |
| *Ar. flavus* | Container |  |  |  | GBP |  |  |  |  |
| *Ar. kesseli* | Container |  |  |  | GBP |  |  |  |  |
| *Ar. malayi* | Container | MG232590 | MG242606 | MG232403 | MG242529 |  |  |  | TLS |
| *Ar. obturbans* | Container |  |  |  |  |  |  | GBP |  |
| *Ar. subalbatus* | Container | MG232591 | MG242607 | MG232402 | MG242530 | GBP | MG232516 | GBP | JPN |
| *Culex quinquefasciatus* | Ground pool, container | GBP | GBP | GBP | GBP | GBP | GBP | GBP |  |
| *Er. quinquevittatus* | Container |  |  | GBP |  |  | GBP |  |  |
| *Hg. equinus* | Container |  |  | GBP | GBP |  | GBP |  |  |
| *Hg. janthinomys* | Container | MG232592 | MG242608 | MG232401 | MG242531 | GBP | MG232517 |  | BRA |
| *Hg. leucocelaenus* | Container | MG232593 |  | MG232400 |  |  | MG232518 | GBP | BRA |
| *Hg. mesodentatus* | Container |  |  |  |  |  |  | GBP |  |
| *Hz. chengi* | Container |  | GBP |  | GBP |  |  |  |  |
| *Hz. discrepans* | Container |  |  |  | GBP |  |  |  |  |
| *Hz. lii* | Container |  |  |  | GBP |  |  |  |  |
| *Hz. menglianensis* | Container |  | GBP |  | GBP |  |  |  |  |
| *Hz. proxima* | Container |  | GBP |  | GBP |  |  |  |  |
| *Hz. reidi* | Container |  | GBP |  | GBP |  |  |  |  |
| *Op. fuscus* | Salt pool |  |  | GBP |  |  | GBP |  |  |
| *Ps. ciliata* | Ground pool | MG232594 | MG242610 | MG232399 | MG242533 |  |  |  | TX, USA |
| *Ps. cingulata* | Ground pool |  |  |  | GBP |  |  |  |  |
| *Ps. columbiae* | Ground pool | MG232595 | MG242611 | MG232398 | MG242534 |  | MG232519 |  | PA, USA |
| *Ps. confinnis* | Ground pool |  |  |  | GBP |  |  |  |  |
| *Ps. cyanescens* | Ground pool | MG232596 | MG242612 | MG232397 | MG242535 |  | MG232520 |  | FL, USA |
| *Ps. ferox* | Ground pool | MG232597 | MG242613 | MG232396 | MG242536 |  | MG232521 | MG232643 | MA, USA |
| *Ps. horrida* | Ground pool | MG232598 | MG242614 | MG232395 | MG242537 |  | MG232522 | MG232644 | PA, USA |
| *Ps. howardii* | Ground pool | MG232599 | MG242615 |  | MG242538 |  | MG232523 | MG232645 | PA, USA |
| *Ps. insularia* | Ground pool |  |  |  | GBP |  |  |  |  |
| *Ps. longipalpus* | Ground pool |  |  |  | GBP |  |  |  |  |
| *Ps. pygmaea* | Ground pool |  |  |  | GBP |  |  |  |  |
| *Ps. signipennis* | Ground pool |  |  |  | GBP |  |  |  |  |
| *Ud. subsimilis* | Container |  | GBP |  | GBP |  |  |  |  |
| *Ve. butleri* | Ground pool |  |  |  | GBP |  |  |  |  |
| *Ve. indica* | Ground pool |  |  |  | GBP |  |  |  |  |
| *Ve. lugubris* | Salt pool |  |  |  | GBP |  |  |  |  |
| *Ve. nobukonis* | Ground pool |  |  |  | GBP |  |  |  |  |
| *Ve. pseudomediofasciata* | Ground pool |  |  |  | GBP |  |  |  |  |
| *Ze. gracilis* | Container |  |  |  | GBP |  |  |  |  |

*Unless indicated, habitat data from Mosquito Taxonomic Inventory or from Reinert et al. 2009 (19).

| Supplemental Table 3. Marker name, length in alignment (including gaps), number of taxa, primer sequences (5) to 3’), and pipeline search terms for markers used in this study. References for primer sequences, where applicable, are given as references to marker name. | | | | |
| --- | --- | --- | --- | --- |
| Marker | Maximum Length | Number of Taxa | Primer sequence | Pipeline Search Terms |
| Small Ribosomal Subunit (18S) | 968 | 81 | F- GTTGAGTCAAATTAAGCCGC  R - CTGCCCTATCAACTATTGATGG | 18S, small subunit |
| Large Ribosomal Subunit (28S) | 951 | 124 | F- GGAGTCGTGTTGCTTGATA  R - CCAGCTACTAGATGGTTCG | 28S, d2, d3, large subunit |
| Arginine Kinase (10) | 714 | 68 | F – GCTTCAAGAAGACCGACAAGCAC  R – GCCATCGTACATCTCCTTGACG | arginine kinase |
| Cytochrome Oxidase Subunit I (COI) (52) | 1401 | 239 | F - GGTCAACAAATCATAAAGATATTGG  R – TAAACTTCAGGGTGACCAAAAAATCA | cox1, coi, cytochrome oxidase subunit I, cytochrome oxidase subunit 1 |
| Cytochrome Oxidase Subunit II (COII) | 679 | 47 | N/A - Only from GenBank | cox2, coii, cytochrome oxidase subunit ii, cytochrome oxidase subunit 2 |
| Enolase (10) | 702 | 71 | F - AGRATYTGGTTGTACTTGGC  R - ATGCAGGAGTTCATGATCCTG | enolase |
| Internal Transcribed Spacer II (ITS2) (23) | 883 | 98 | F – TGTGAACTGCAGGACACATG  R - TCACACATTATTTGAGGCCTAC | ITS2, internal transcribed spacer 2 |

| Supplemental Table 4: The best fitting partition scheme, according to PartitionFinder 2. | | |
| --- | --- | --- |
| Partition Number | Region | Model |
| 1 | 18S | GTR+G |
| 2 | 28S | GTR+G |
| 3 | ITS2 | GTR+G |
| 4 | 1st Codon, Arginine Kinase | GTR+G |
| 5 | 2nd Codon, Arginine Kinase | GTR+G |
| 6 | 3rd Codon, Arginine Kinase | GTR+G |
| 7 | 1st Codon, Enolase | GTR+G |
| 8 | 2nd Codon, Enolase | GTR+G |
| 9 | 3rd Codon, Enolase | GTR+G |
| 10 | First Codon, COI | GTR+G |
| 11 | Second Codon, COI | GTR+G |
| 12 | First Codon, COII | GTR+G |
| 13 | Second Codon, COII | GTR+G |
| 14 | Third Codon, COI and COII | GTR+G |

| Supplemental Table 5. The fit of different transition rates for ancestral state reconstruction from Bayesian stochastic character mapping. | | | |
| --- | --- | --- | --- |
| Model | AICCc | ΔAICc | Weight |
| ER | 309.99 | 0 | 0.96 |
| SYM | 316.80 | 6.81 | 0.03 |
| ARD | 322.49 | 12.49 | <0.01 |

| Supplemental Table 6: Select morphological characters, and their weighting and coordinates for dimension 1. Description of characters from Reinert et al. 2009 (19) | | | | |
| --- | --- | --- | --- | --- |
| Character Number | State | Character Definition (Given State) | Weight on Dimension 1 | Coordinate on Dimension 1 |
| 7 | 0 | Spicules on antenna, absent | 2.2 | 1.15 |
| 29 | 0 | Seta 2P on thorax, single | 0.14 | 0.18 |
| 41 | 0 | Seta 4M on thorax single, not branched. | 2.54 | 1.38 |
| 101 | 1 | Seta 4d-X of ventral brush not plumose, single or two branched. | 2.21 | 1.11 |
| 7 | 0 | Spicules on antenna, present | 1.01 | -0.53 |
| 29 | 1 | Seta 2P on thorax, branched. | 0.58 | -0.82 |
| 41 | 1 | Seta 4M on thorax, branched. | 1.01 | -0.52 |
| 101 | 2 | Seta 4d-X of ventral brush plumose. | 1.25 | -0.61 |
| *Weight of a character is the influence on a dimension (out of all characters), while a coordinate is directional the the influence on final dimension 1 scores that the trait has. | | | | |

| Supplemental Table 7. Multivariate Analyses of Variance on five morphological dimensions and habitat specialization | | | | | | | | |
| --- | --- | --- | --- | --- | --- | --- | --- | --- |
| Null Model* | Residual Df | Df | Pillai-Barlett | Approx F | Num Df | Den Df | P | P (Simulated Null) |
| F-stat | 113 | 8 | 1.1281 | 4.1154 | 40 | 565 | 1.68E-14 | N/A |
| BM |  |  |  |  |  |  |  | <0.001 |
| OU |  |  |  |  |  |  |  | <0.001 |
| EB |  |  |  |  |  |  |  | <0.001 |
| OUM*** |  |  |  |  |  |  |  | 0.42 |
| *Null model refers either to the F-distribution, or to the model under which the null distribution was simulated, based on parameter estimates from the original data. The first row is a standard MANOVA. | | | | | | | | |
| **Note that MANOVA test statistics and degrees of freedom remain the same, regardless of the null distribution, and so are not shown | | | | | | | | |
| ***This is the best-scoring OUM model with the stochastic character map shown in Figure 1. | | | | | | | | |

| Supplemental Table 8: A model comparison of the fit of different models of evolution to our morphological trait data from | | | |
| --- | --- | --- | --- |
| Model | AICCc | ΔAICc | Weight |
| OUM | -54.85 | 0 | 0.86 |
| EB | -49.88 | 4.97 | 0.07 |
| BM | -49.72 | 5.13 | 0.06 |
| OU1 | -8.99 | 45.86 | 9.50E-11 |
