# Supplementary material for: From ground pools to treeholes: convergent evolution of habitat and phenotype in Aedes mosquitoes
Source: BMC Evol Biol. 2017 Dec 19;17:262. doi: 10.1186/s12862-017-1092-y (PMC5735545; doi:10.1186/s12862-017-1092-y)
Supplement: Supplementary file 2 — supplement_textv1. Supplemental methods, results, and discussion. This document provides additional details on our methods, as well as consideration of our phylogenetic results and a discussion of their significance in this supplemental material. (DOCX 22 kb) [file 12862_2017_1092_MOESM2_ESM.docx]

**SUPPLEMENTAL METHODS**

*GenBank Datamining.* To augment our sequencing data with publicly available sequence data, we implemented a GenBank pipeline from the R package metaptera (available from the author at https://github.com/heibl/megaptera). megaptera is an R package designed to build supermatrices of multiple markers for a given taxonomic group and marker set. megaptera downloads all sequences within the specified group of taxa for the given markers; it then compares sequences to a reference (usually generated in the pipeline, but optionally supplied by the user) and discards sequences with pairwise nucleotide distance above a threshold of similarity (in our case 0.75). We defined our ingroup of interest as the Aedini. We provided reference sequences to the pipeline based on consensus sequences for all markers. For 18S, 28S, ITS2, enolase, and arginine kinase, these consensus sequences were from alignments from sequences we generated. However, for COII and COI, we extracted sequences from the mitochondrial genomes of *Aedes (Stegomyia) albopictus* (GenBank Accession NC_006817), *Aedes (Stegomyia) aegypti* (NC_010241), *Haemagogus janithomys* (NC_028025), *Aedes (Ochlerotatus) vigilax* (NC_027494), and *Aedes (Rampamyia) notoscriptus* (NC_025473). We aligned these sequences in Geneious with MAFFT, and generated consensus sequences for COI and COII. We then added our consensus sequences to the database table generated by the pipeline, and resumed the pipeline. Finally, megaptera builds consensus sequences per species per marker using MAFFT. These consensus sequences are then aligned and can be output on phylip format, or run through additional masking steps (not used in this study) prior to export. Sequences were retrieved in July 2014, and sequence alignments were periodically updated until June 2017. For each sequence from our pipeline, we used megablast in Geneious to evaluate sequence identity to other sequences on GenBank. We excluded any sequences from our analysis where a BLAST hit was not either a member of the same subgenus (if multiple sequences from that subgenus were available) or another closely related (e.g. within the Aedini) mosquito.

**SUPPLEMENTAL RESULTS AND DISCUSSION**

*Sequence quality filtering.* During the process of BLASTing all sequences from our pipeline, we noted that COI sequences from *Aedes (Phagomyia) cogili*, *Aedes (Stegomyia) malayensis*, *Aedes (Mucidus) aurantius*, and *Haemagogus lucifer* resolved to taxa other than members of their subgenus. For *Haemagogus lucifer*, BLAST results suggested affinity with a mosquito outside of the Aedini, and preliminary phylogenetic results showed *Hg. lucifer* clustering with the *Culex quinquefasciatus* outgroup, despite all other *Haemgogus* resolving as monophyletic and within the Aedini. Thus, we removed this taxon. For *Aedes (Phagomyia) cogili*, BLAST searches suggested a high identity (>99%) to COI sequences from *Aedes (Fredwarsius) vittatus*, a taxon for which we had multiple markers. Moreover, despite all other members of the subgenus resolving as monophyletic deep within Clade B, this taxon resolved with *Ae. (Fredwarsius) vittatus* in Clade A. For *Aedes (Stegomyia) malayensis*, there were several BLAST hits that were nearly identical (>99%) to *Aedes (Bothaella) manhi*, and in preliminary analyses, *Aedes (Stegomyia) malayensis* did cluster with the subgenus *Bothaella*, violating monophyly of both *Stegomyia* and *Bothaella*. For *Aedes (Mucidus) aurantius*, COI sequence identity was less than 90% for any other mosquito, despite COI sequences for two other members of its subgenus being available; moreover, preliminary phylogenetic analyses suggested the taxa was not related to either other *Mucidus* in the analysis. Inclusion or exclusion of this taxa did not change the results of comparative analyses, so we chose to exclude these taxa, because we could not be certain that the GenBank sequences were from these taxa.

*Comparative analyses with our phylogeny of three or more markers.* Our results for our comparative analyses with our maximum likelihood phylogeny of 103 taxa (average ungapped nucleotide coverage of 3550) represented by three or more markers were qualitatively the same as for our analyses with all 260 taxa. For instance, we recovered strong support for a ground pool dwelling ancestor of both the Aedini and *Aedes*, with posterior probability of this habitat specialization of 0.96 and 0.95, respectively (Supplemental Figure 7). Once we pruned our phylogeny of three or more markers to only those taxa for which we had morphological data for comparative analyses, we had 64 taxa for which we had habitat, morphology, and phylogenetic data. A phylogenetic MANOVA on this subset of our data rejected the null models under BM, EB, and OU1 (P<0.001). Finally, as with our complete data set, a multivariate multi-optima OU model was the best fit for the data. That our comparative analyses involving this reduced data set, which used only 22 taxa from GenBank and only those with multiple markers to compliment the 81 we sequenced ourselves, also supports identical conclusions of our complete analysis suggests that our complete analysis is not bias due to the inclusion of taxa with low marker coverage from GenBank.

*Ancestral state reconstruction with an uncalibrated ML topology.* In agreement with the results of our ancestral state reconstructions using our calibrated ML phylogeny, we found strong support for an ancestral ground pool habitat specialization in the Aedini, and convergent evolution of container specialization using both stochastic character mapping and ancestral character estimation (Fig. S9, Fig S10).

*The maximum likelihood phylogeny of the Aedini and its implication for taxonomy.* Our maximum likelihood phylogeny is consistent with the previous molecular phylogenies involving aedine taxa. For instance, although sampling only 19 *Aedes* from the Northeastern United States, Shepard et al. (22) also recovered evidence of the two clades we recovered here, save that they lacked samples from non-*Aedes* genera in the aedine, other than *Psorophora*. Both Besansky and Faye (24) and Reidenbach et al. (10) used very few aedines – 4 and 6, respectively – but found that *Aedes* was not monophyletic, as *Aedes (Ochlerotatus) triseriatus* was more closely related to *Haemagogus equinus* in both analyses than it was to *Aedes (Stegomyia)* mosquitoes, consistent with our results.

Strikingly, our results are also consistent with morphological observations on the sexual anatomy of adult males and other morphological features that were originally used to divide the genus *Aedes* into two genera by the elevation of *Ochlerotatus* (16)*.* This taxonomic action sorted all *Aedes* taxa into one of two genera: *Aedes* sensu Reinert 2000 and *Ochlerotatus* sensu Reinert 2000 (16), which conform exactly to our recovered clades, Clade A and Clade B, as well as to those clades recovered by other authors (22) . Following this action, however, Reinert et al. (16-19) took additional taxonomic action in a series of studies based on ever increasing numbers of samples and morphological characters, culminating in the elevation of 72 subgenera from *Aedes* to generic status (19). However, although our results agree with quantitative cladistics demonstrating the lack of monophyly of *Aedes* (19), the particular relationships reflected by our molecular phylogeny differ substantially from those derived from morphological datasets. Here we focus predominantly on a comparison between our results those of Reinert et al. (19) (8); although Wilkerson et al. (6) used the same dataset as Reinert et al., they failed to find strong support for internal branches in general and so it is difficult to make meaningful comparisons with their results. Disagreement between our results and morphology-based analyses is ultimately unsurprising, as we here demonstrated that a large portion of the characters used in the aforementioned analyses are convergent. Interestingly, we recover some of the same groups as Reinert et al., particularly when dealing with only ground pool breeding mosquitoes. Within Clade A, we recovered a clade comprised predominantly of ground pool breeding mosquitoes, made up of the genus *Verallina* and the *Aedes* subgenera *Aedes*, *Paraedes*, *Edwardaedes*, and *Neomonlicon*, a clade similarly recovered by Reinert et al. In Clade B, we find that *Aedes (Arcartomyia)* is related to *many Aedes (Ochlerotatus)*, as did Reinert et al.

On the other hand, we find markedly different relationships between container breeding Aedini. For instance, in Clade A, we find that all *Aedes* subgenera sensu Reinert 2000 – including the subgenera *Stegomyia*, *Aedimorphus*, *Aedes*, *Bothaella*, and others – are in a clade sister to most non-*Aedes* genera, namely *Armigeres*, *Eretmapodites*, *Udaya*, *Heizmannia*, and *Zeugnomyia*. All of these non-*Aedes* genera are container breeding, and so too are *Stegomyia*. That all *Aedes* sensu Reinert 2000 are in a clade contrasts with the finding of Reinert et al. (19), wherein *Stegomyia* shared a more recent common ancestor with these non-Aedes genera (and some other *Aedes* subgenera) than with the majority of *Aedes* sensu Reinert 2000 subgenera (e.g. the above named subgenera). Moreover, within Clade B, where convergence to container breeding occurred multiple times, we recover markedly different relationships by and large. For instance, we recovered *Haemagogus* as sister to Aedes (*Howardina’*, and within a clade of nearctic and neotropical Aedes (*Ochlerotatus)* mosquitoes, which differed markedly from Reinert et al. (19), where *Haemagogus* formed a clade Aedes subgenera *Downsiomyia*, *Danielsia*, and *Finlaya*, all subgenera predominantly found in the oriental region, Australia, and parts of the Palearctic; *Hamaegogus*, however, is found exclusively in the Neotropics, with one species found in southern North America.

Ultimately, although our results differ substantially from many of those relationships recovered by Reinert et al., we ultimately find, as they did, that *Aedes* is not monophyletic, and that this violation stems from multiple aedine genera. Indeed, although Wilkerson et al. demoted the elevated *Aedes* subgenera to their current status, as they were unable to recover well-supported violations of monophyly of the genus *Aedes*, they were also unable to recover strong evidence of *Aedes* as monophyletic. However, because our analysis relied so heavily on GenBank taxa with limited coverage, our results may be insufficient to justify taxonomic action. Instead, our results highlight the pressing need for additional robust (in terms of higher marker coverage) and densely sampled (in terms of species) phylogenies, such that taxonomic action could stem from the weight of morphological evidence from as well molecular phylogenies.
