# Supplementary figures and images for: From ground pools to treeholes: convergent evolution of habitat and phenotype in Aedes mosquitoes
Source: BMC Evol Biol. 2017 Dec 19;17:262. doi: 10.1186/s12862-017-1092-y (PMC5735545; doi:10.1186/s12862-017-1092-y)

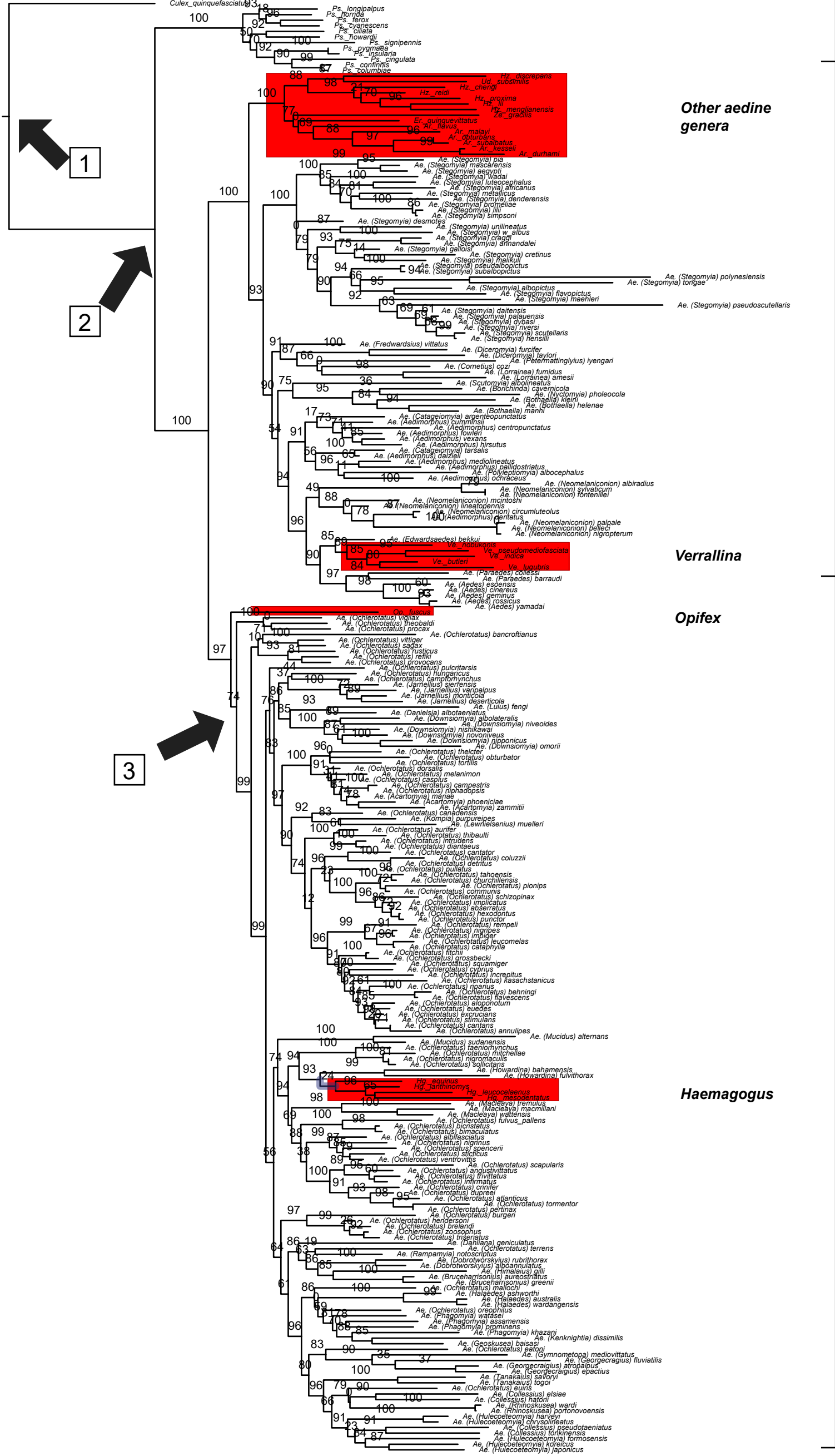

Supplement: Supplementary file 3 — Figure S1. The maximum likelihood phylogeny from RAxML with all taxa included. Genera that violate the monophyly of Aedes are highlighted in red. The tree has been rooted leading to the branch for Culex quinquefasciatus. Scale is in substitutions per site. Calibration points used in this study have been indicated with numbered arrows. Details on calibration points are given in our methods. 1: The common ancestor of Culex and Aedes between 226.22 and 172.28 MYA. 2: The common ancestor of all aedine mosquitoes between 155.71 and 90.18 MYA. 3: The common ancestor of all Aedes (Ochlerotatus) species, with a minimum age of 33.9 MYA and a maximum age of 94.29 MYA. (PDF 401 kb) [file 12862_2017_1092_MOESM3_ESM.pdf]

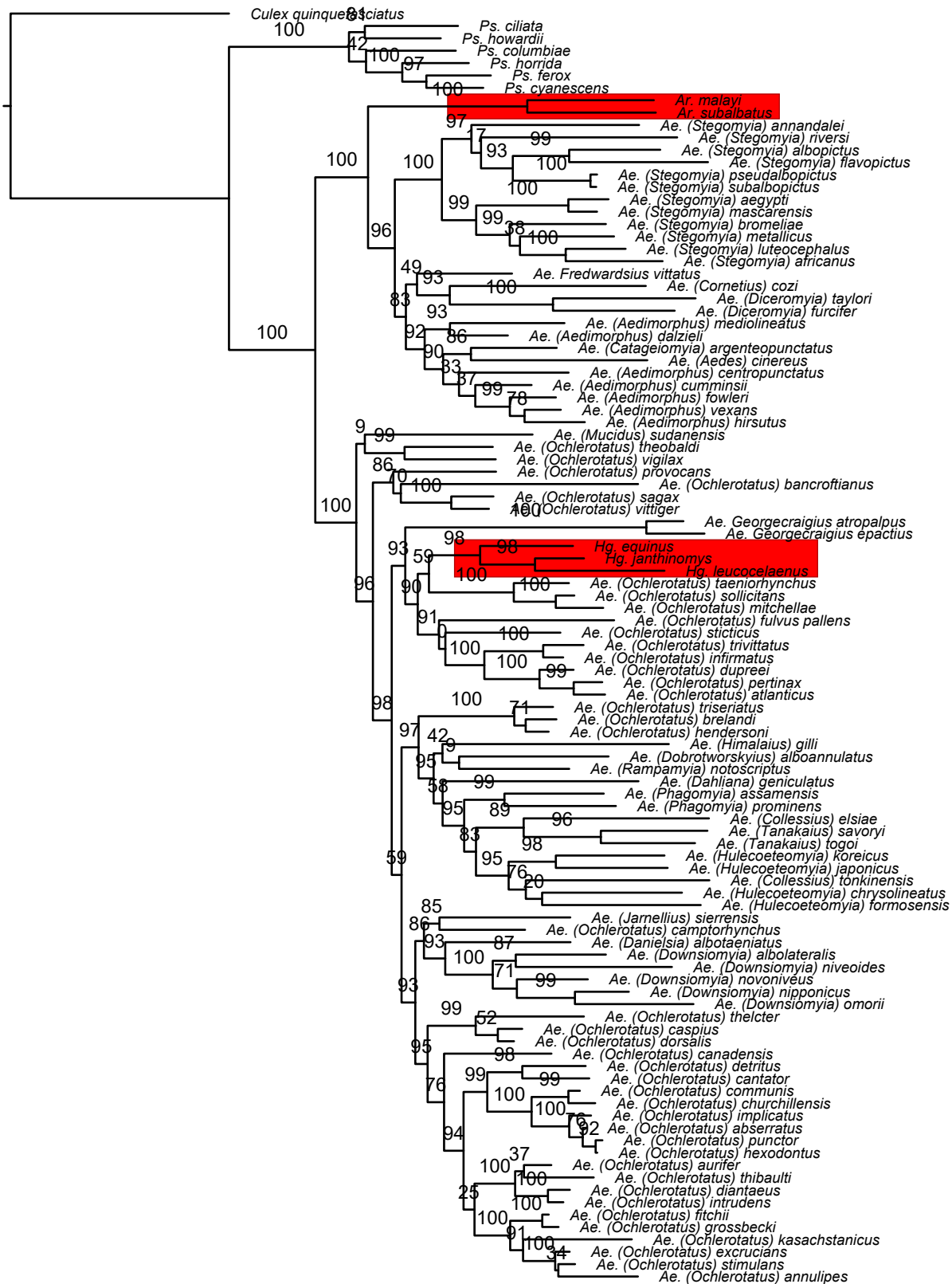

Supplement: Supplementary file 4 — Figure S2. The maximum likelihood phylogeny from an alignment containing only taxa with three or more markers. Scale is in substitutions per site. We still recover Clade A and B from our full analysis in this analysis, containing 104 taxa with high marker coverage. Genera that violate the monophyly of Aedes are highlighted in red. (PDF 217 kb) [file 12862_2017_1092_MOESM4_ESM.pdf]

Habitat:

- 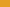 Ground Pool
- 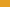 Salt Pool
- 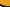 Rock Pool
- 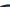 Container
- 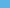 Crab Hole

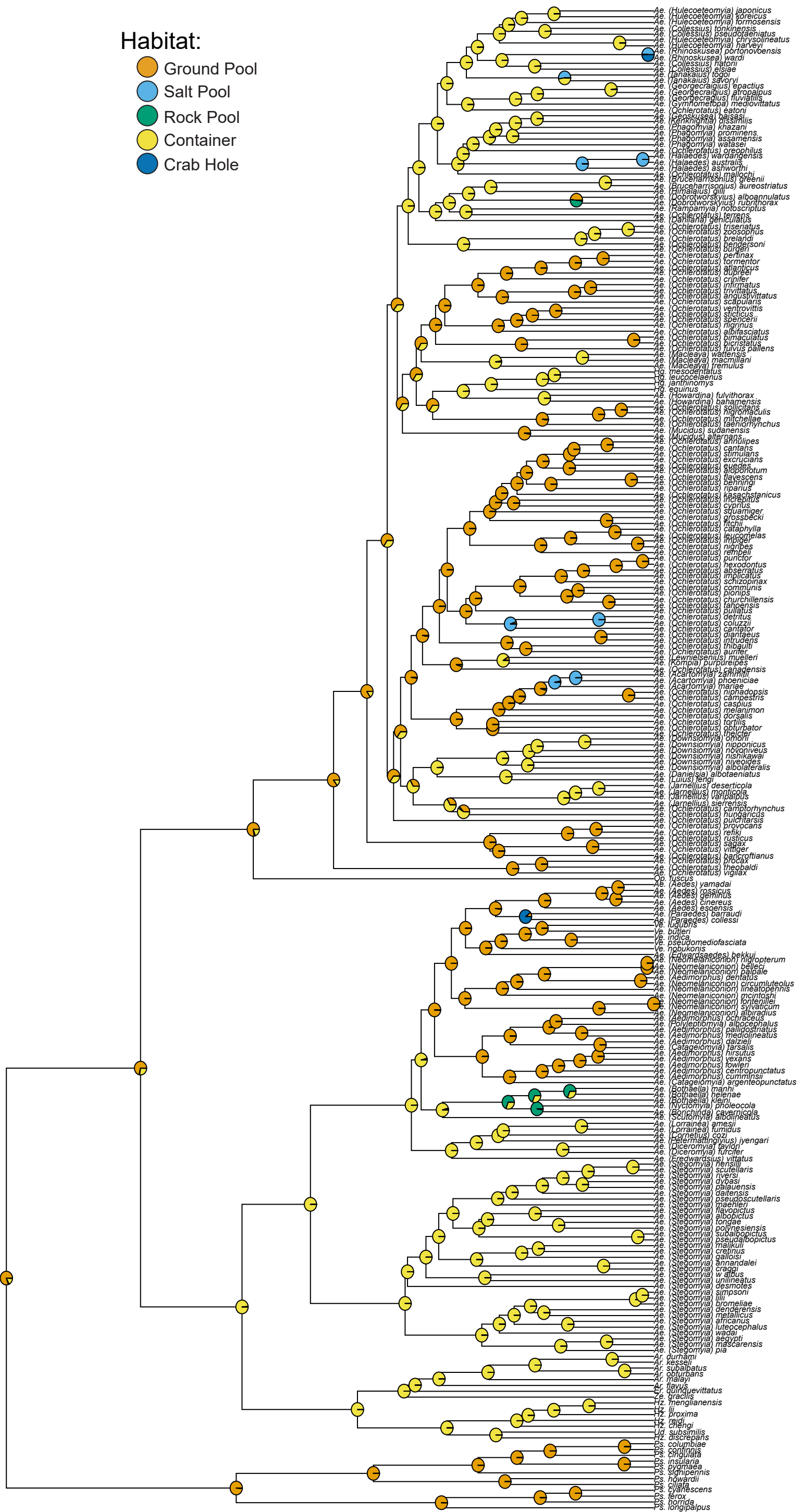

Supplement: Supplementary file 5 — Figure S3. Maximum likelihood ancestral character reconstruction of discrete characters suggests that ground pool dwelling was ancestral in the Aedini and in Aedes. The time-calibrated maximum likelihood phylogeny from our analysis of the Aedini showing putative ancestral character states from our maximum likelihood analysis of discrete characters is shown here. Size of pie slices shows the likelihood of a habitat type. (PDF 1.27 mb) [file 12862_2017_1092_MOESM5_ESM.pdf]

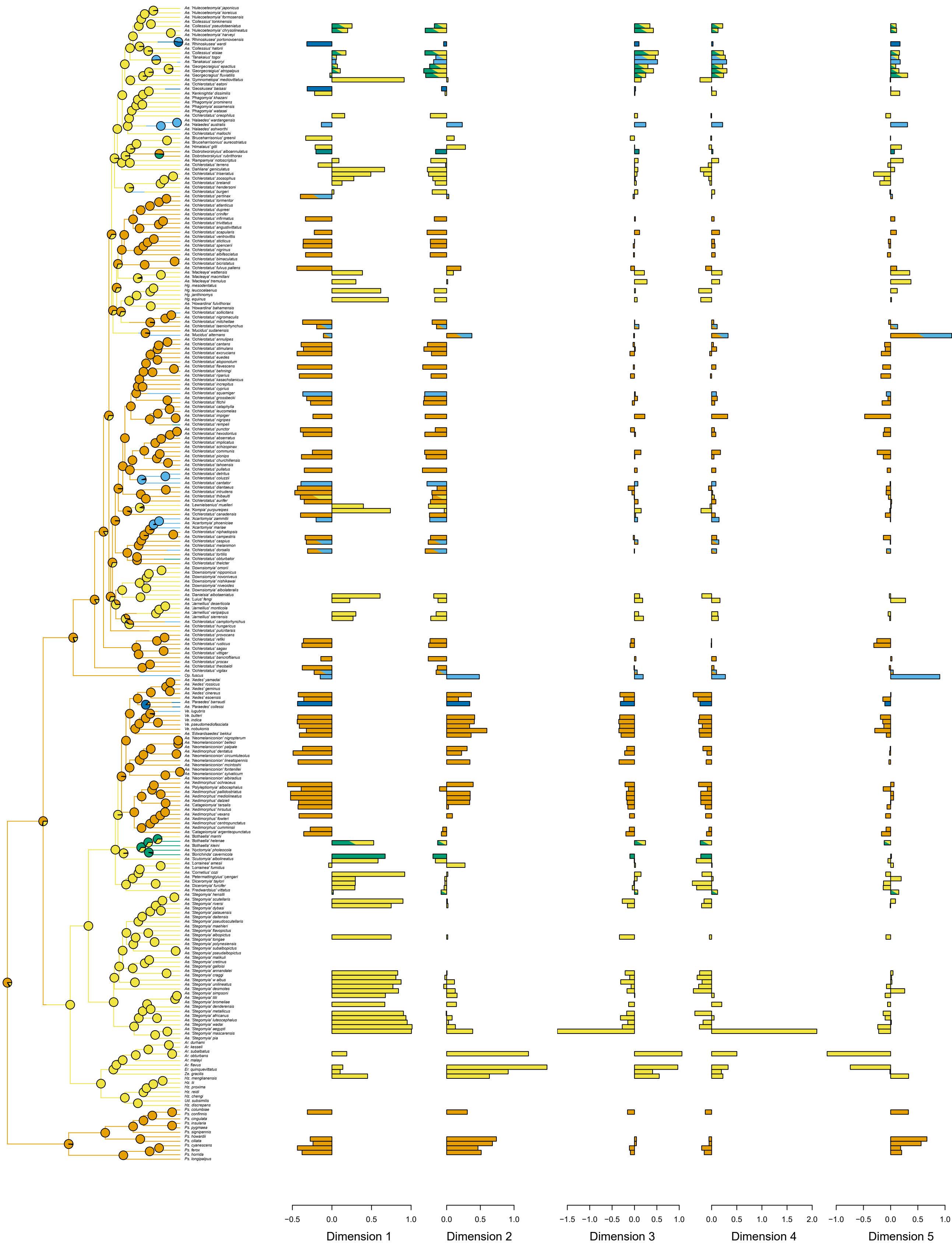

Supplement: Supplementary file 7 — Figure S5. The time-calibrated maximum likelihood phylogeny from our analysis of the Aedini showing habitat transitions and putative ancestral character states from one of our stochastic character maps, along with the five dimensions from our multiple correspondence analysis, colored by habitat preference. (PDF 673 kb) [file 12862_2017_1092_MOESM7_ESM.pdf]

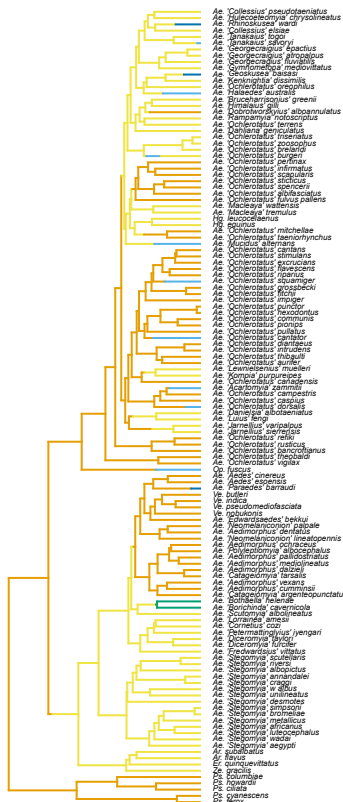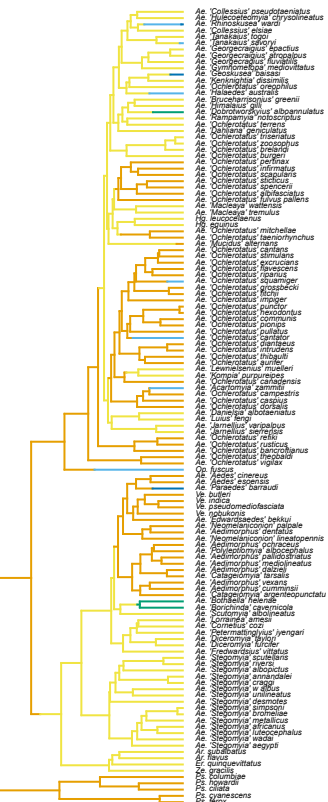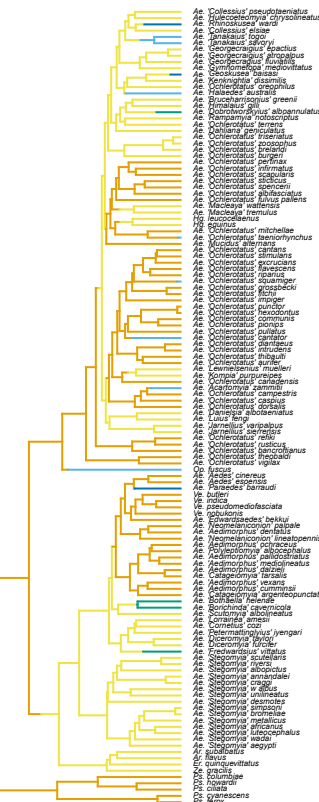

Supplement: Supplementary file 8 — Figure S6. The three stochastic character maps representing the best-scoring OUM models 1, 2, and 3 from Table 3. Full model parameters are given in Table S8. (PDF 517 kb) [file 12862_2017_1092_MOESM8_ESM.pdf]

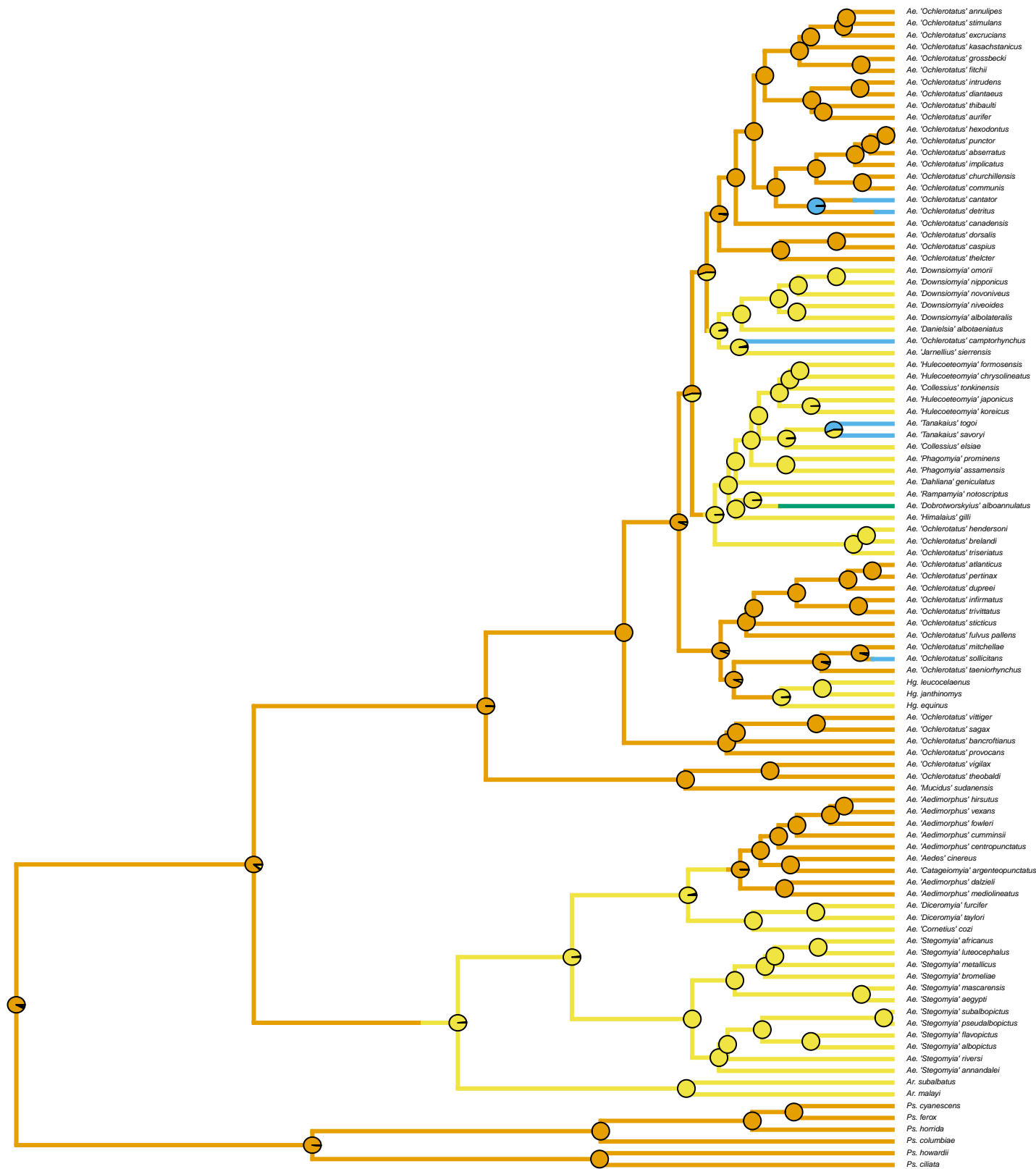

Supplement: Supplementary file 9 — Figure S7. One stochastic character map from our Bayesian stochastic character map of our reduced data set containing 103 taxa for which we had marker coverage of three or more. As in the case of our complete analysis, we find that ground pool dwelling was likely ancestral in both the Aedini as a whole and in the genus Aedes. (PDF 41 kb) [file 12862_2017_1092_MOESM9_ESM.pdf]

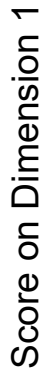

Habitat:

- 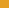 Ground Pool
- 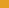 Salt Pool
- 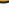 Rock Pool
- 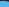 Container
- 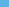 Crab Hole

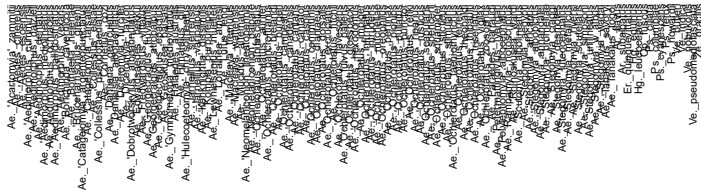

Taxa

Supplement: Supplementary file 10 — Figure S8. Mean simulated values along dimension 1 (black dots) with two standard deviations (whiskers). Real values on dimension 1 shown as colored circles. Real values fall within two standard deviations of all simulated values. (PDF 307 kb) [file 12862_2017_1092_MOESM10_ESM.pdf]

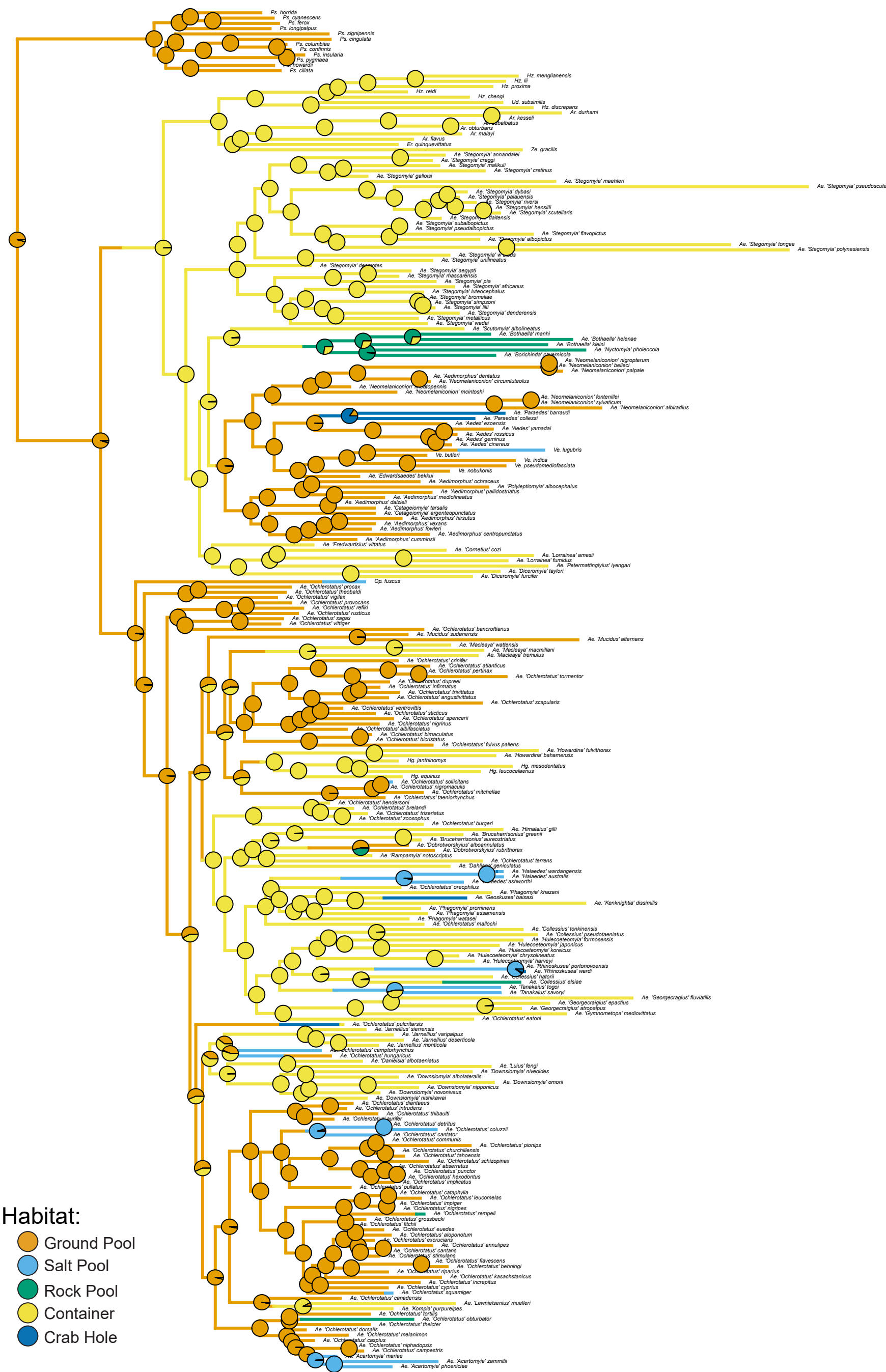

Supplement: Supplementary file 11 — Figure S9. One stochastic character map from our stochastic character map on our untransformed maximum likelihood phylogeny. As in the case of our complete analysis, we find that ground pool dwelling was likely ancestral in both the Aedini as a whole and in the genus Aedes. Size of pie slices shows the posterior probability of a habitat type. (PDF 609 kb) [file 12862_2017_1092_MOESM11_ESM.pdf]

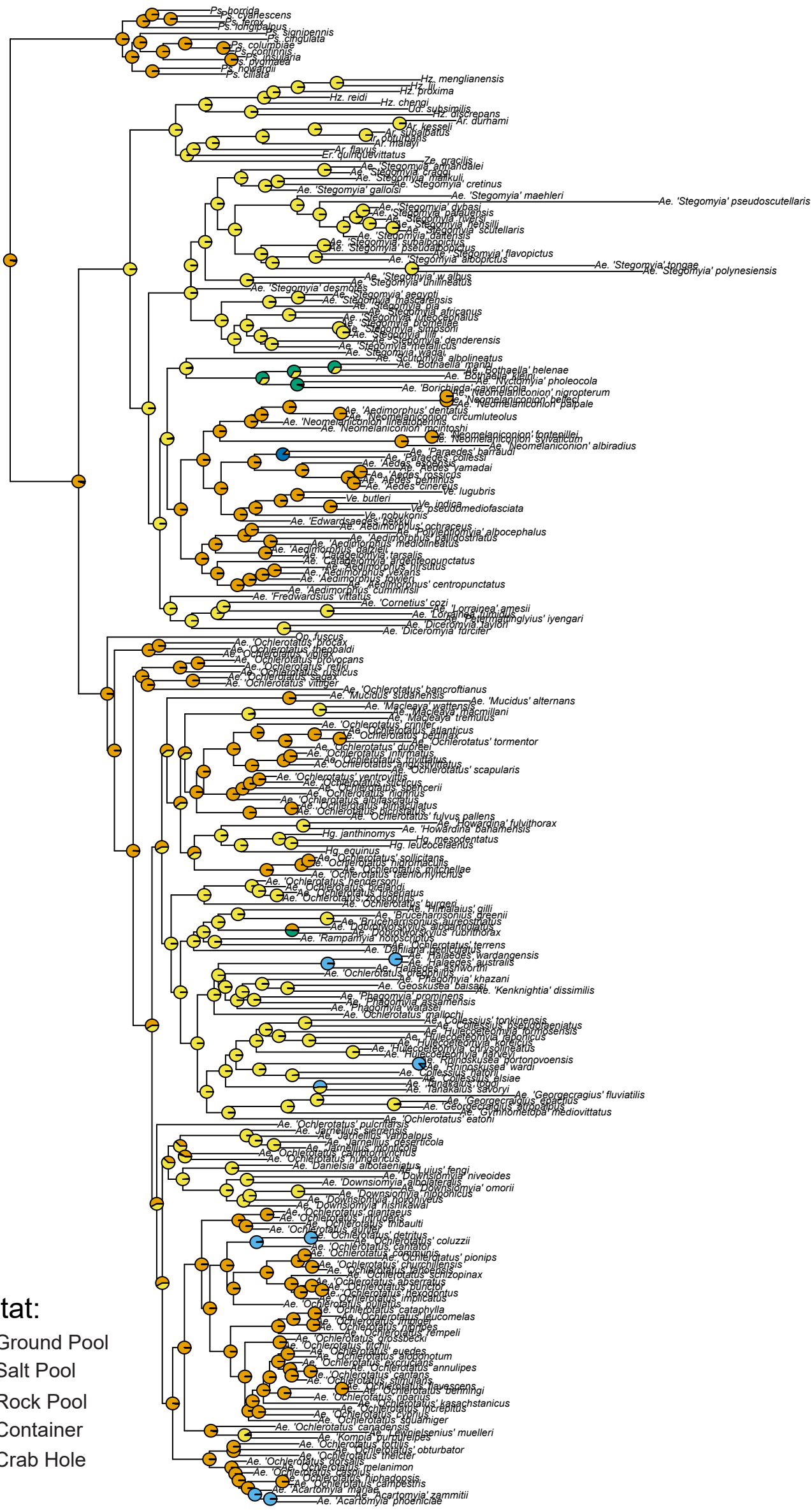

Habitat:

- Ground Pool
- Salt Pool
- Rock Pool
- Container
- Crab Hole

Supplement: Supplementary file 12 — Figure S10. The maximum likelihood ancestral character reconstruction of discrete characters along our untransformed maximum likelihood phylogeny suggests that ground pool dwelling was ancestral in the Aedini and in Aedes. Size of pie slices shows the likelihood of a habitat type. (PDF 1.25 mb) [file 12862_2017_1092_MOESM12_ESM.pdf]
