# Supplementary material for: From ground pools to treeholes: convergent evolution of habitat and phenotype in Aedes mosquitoes
Source: BMC Evol Biol. 2017 Dec 19;17:262. doi: 10.1186/s12862-017-1092-y (PMC5735545; doi:10.1186/s12862-017-1092-y)
Supplement: Supplementary file 6 — Figure S4. A comparison of four larval mounts, two from container dwelling mosquitoes, Aedes (Ochlerotatus) triseriatus and Aedes (Stegomyia) albopictus and two from ground pool dwelling mosquitoes Aedes (Ochlerotatus) excrucians and Aedes (Aedimorphus) vexans. Aedes (Stegomyia) albopictus and Aedes (Aedimorphus) vexans are in Clade A, while Aedes (Ochlerotatus) triseriatus and Aedes (Ochlerotatus) excrucians are in Clade B. (PDF 8.19 mb) [file 12862_2017_1092_MOESM6_ESM.pdf]

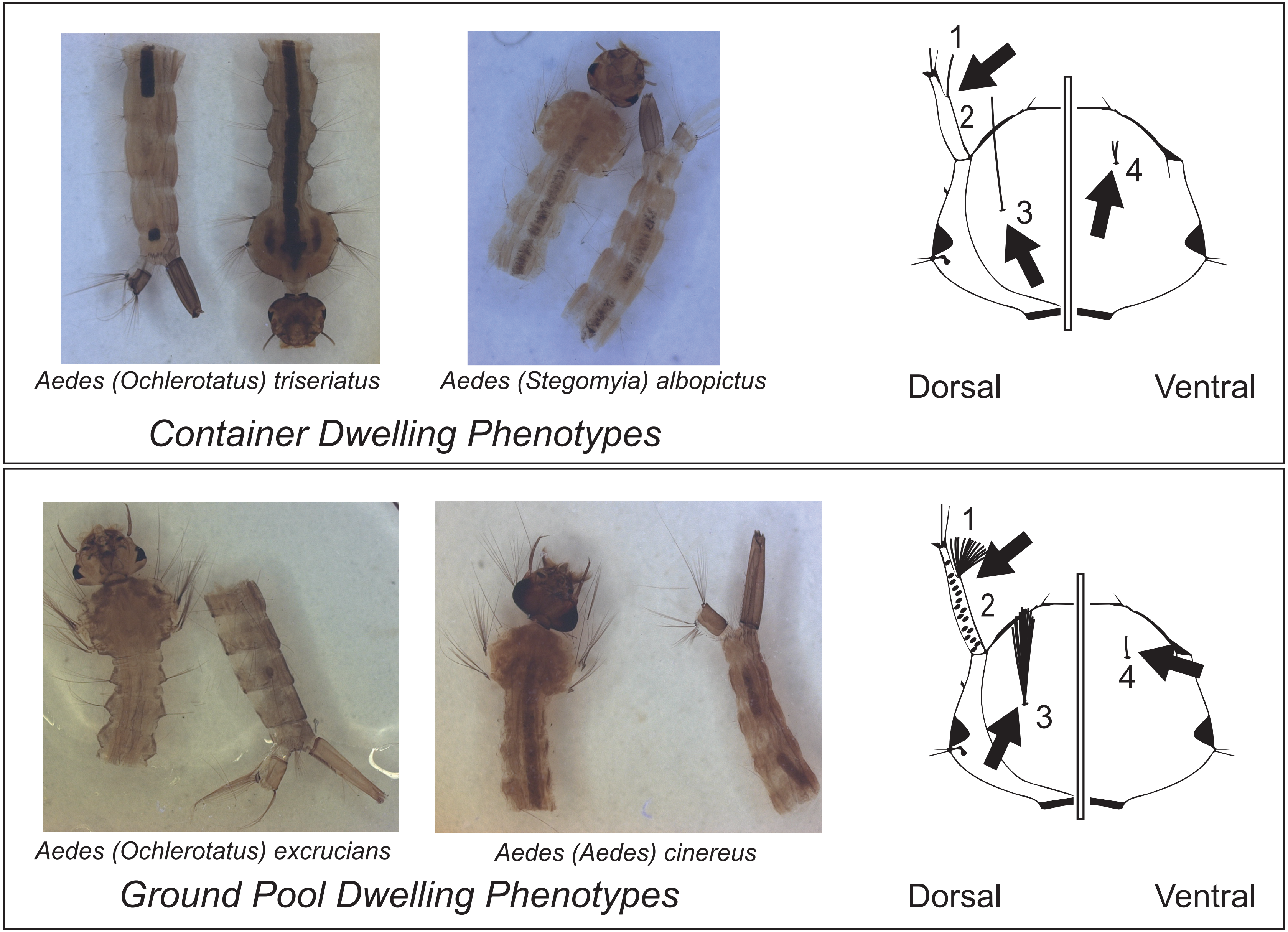
Aedes (Ochlerotatus) triseriatus
Aedes (Stegomyia) albopictus
1
2
3
4
Dorsal
Ventral
Container Dwelling Phenotypes
Aedes (Ochlerotatus) excrucians
Aedes (Aedes) cinereus
1
2
3
4
Dorsal
Ventral
Ground Pool Dwelling Phenotypes
